# First-Row Transition Metal Complexes Incorporating the 2-(2′-pyridyl)quinoxaline Ligand (pqx), as Potent Inflammatory Mediators: Cytotoxic Properties and Biological Activities against the Platelet-Activating Factor (PAF) and Thrombin

**DOI:** 10.3390/molecules28196899

**Published:** 2023-10-01

**Authors:** Antigoni Margariti, Vasiliki D. Papakonstantinou, George M. Stamatakis, Constantinos A. Demopoulos, Christina Machalia, Evangelia Emmanouilidou, Gregor Schnakenburg, Maria-Christina Nika, Nikolaos S. Thomaidis, Athanassios I. Philippopoulos

**Affiliations:** 1 Laboratory of Inorganic Chemistry, Department of Chemistry, National and Kapodistrian University of Athens, Panepistimiopolis Zografou, 15771 Athens, Greece; antmargariti@gmail.com; 2 Laboratory of Biochemistry, Department of Chemistry, National and Kapodistrian University of Athens, Panepistimiopolis Zografou, 15771 Athens, Greece; papakonstantinou.v@gmail.com (V.D.P.); stamatakisgeo@gmail.com (G.M.S.); demopoulos@chem.uoa.gr (C.A.D.); cmachali@chem.uoa.gr (C.M.); eemman@chem.uoa.gr (E.E.); 3 Institut für Anorganische Chemie, Rheinische Friedrich-Wilhelms-Universität Bonn, Gerhard-Domagk-Straße 1, D-53121 Bonn, Germany; gschnake@uni-bonn.de; 4 Laboratory of Analytical Chemistry, Department of Chemistry, National and Kapodistrian University of Athens, Panepistimiopolis Zografou, 15771 Athens, Greece; nikamar@chem.uoa.gr (M.-C.N.); ntho@chem.uoa.gr (N.S.T.)

**Keywords:** metal complex, first-row transition metals, 2-(2′-pyridyl)quinoxaline, Platelet-Activating Factor, thrombin, PAF-inhibitors, thrombin-inhibitors, lipid mediators, inflammation, cytotoxicity

## Abstract

Inflammatory mediators constitute a recently coined term in the field of metal-based complexes with antiplatelet activities. Our strategy targets Platelet-Activating Factor (PAF) and its receptor, which is the most potent lipid mediator of inflammation. Thus, the antiplatelet (anti-PAF) potency of any substance could be exerted by inhibiting the PAF-induced aggregation in washed rabbit platelets (WRPs), which internationally is a well-accepted methodology. Herein, a series of mononuclear (*mer*-[Cr(pqx)Cl_3_(H_2_O]) (**1**), [Co(pqx)Cl_2_(DMF)] (**2**) (DMF = *N*,*N*′-dimethyl formamide), [Cu(pqx)Cl_2_(DMSO)] (**3**) (DMSO = dimethyl sulfoxide), [Zn(pqx)Cl_2_] (**4**)) and dinuclear complexes ([Mn(pqx)(H_2_O)_2_Cl_2_]_2_ (**5**), [Fe(pqx)Cl_2_]_2_ (**6**) and [Ni(pqx)Cl_2_]_2_ (**7**)) incorporating the 2-(2′-pyridyl)quinoxaline ligand (pqx), were biologically evaluated as inhibitors of the PAF- and thrombin-induced aggregation in washed rabbit platelets (WRPs). The molecular structure of the five-co-ordinate analog (**3**) has been elucidated by single-crystal X-ray diffraction revealing a trigonal bipyramidal geometry. All complexes are potent inhibitors of the PAF-induced aggregation in WRPs in the micromolar range. Complex (**6**) displayed a remarkable in vitro dual inhibition against PAF and thrombin, with IC_50_ values of 1.79 μM and 0.46 μM, respectively. Within the series, complex (**5**) was less effective (IC_50_ = 39 μM) while complex (**1**) was almost 12-fold more potent against PAF, as opposed to thrombin-induced aggregation. The biological behavior of complexes **1**, **6** and **7** on PAF’s basic metabolic enzymatic pathways reveals that they affect key biosynthetic and catabolic enzymes of PAF underlying the anti-inflammatory properties of the relevant complexes. The in vitro cytotoxic activities of all complexes in HEK293T (human embryonic kidney cells) and HeLa cells (cervical cancer cells) are described via the 3-(4,5-dimethylthiazol-2-yl)-2,5-diphenyl tetrazolium bromide (MTT) assay. The results reveal that complex **3** is the most potent within the series.

## 1. Introduction

Over the last decades, metal-based drugs have constituted a long-standing research tool against a variety of diseases that are responsible for the increased number of deaths worldwide [1]. Apparently, research in the field of inorganic medicinal chemistry has been dominated by the study of the anti-cancer properties of transition metal complexes, mainly platinum drugs [2,3]. In the meantime, new strategies [4] have appeared to overcome the side-effects acquired with the inherent acute toxicity of cisplatin and several platinum-containing derivatives. Other issues to overcome were those related to the observed drug resistance, upon administration of the pharmacophore (for the case of anticancer agents) and lack of activity against several types of cancer [5]. As a result, a series of new metal-based drugs with other metal ions and with great potencies such as ruthenium [6], gold [7,8], cobalt [9], copper [10], rhodium [11,12], and many others have been developed [13].

Despite the extended research in the field, the effect of metal ions and metal-based complexes in other activities such as cardiovascular diseases and thrombosis, which are the leading causes of death worldwide, have been described to a lesser extent [14].

**Figure 1:**
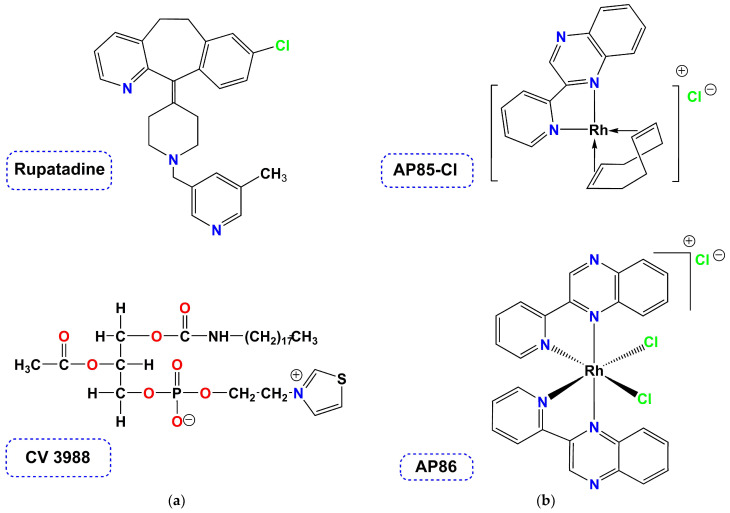
(**a**) Organic antiplatelet agents (PAF-inhibitors); (**b**) Structures of known *inflammatory mediators* (metal-based analogs).

Targeting platelets, thrombin and other inflammatory mediators is a realistic means to prevent acute thromboembolic artery occlusions in cardiovascular diseases and treat chronic inflammatory diseases [15]. Thus far the great majority of compounds with antiplatelet activities are of organic origin (organic small molecules, Figure 1a).

This for example expands to a wide variety of substances including natural products and synthetic organic molecules [16]. Considering the wide range of platelet agonists current antiplatelet drugs that are used clinically, can be classified into four different classes, including (i) cyclooxygenase 1 (COX1) inhibitors (ii) inhibitors of the ADP P2Y_12_ (ADP = adenosine diphosphate receptor) (iii) PAR1 (proteinase-activated receptor) antagonists and (iv) GPIIb/IIIa (GP = glycoprotein) inhibitors [15]. The development of safer, next-generation antiplatelet drugs is of high priority. Recently a sub-topic emerged, the so-called metal-based antiplatelet agents (Figure 1b), revealing the great potencies of coordination compounds and organometallic complexes towards this goal [17,18].

**Figure 2:**
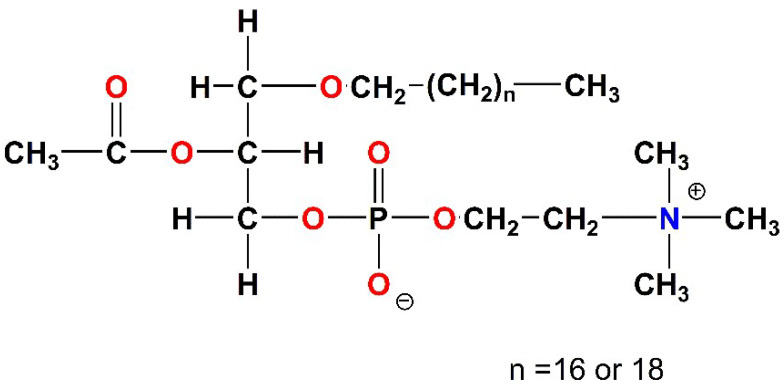
The molecular structure of the Platelet-Activating Factor (PAF).

To achieve this aim (new antiplatelet agents), we have focused on the Platelet-Activating Factor (PAF) and its receptor, as the lead biological target (Figure 2), which is a potent lipid inflammatory mediator [19,20,21]. Our approach is based on the inhibition of the PAF-induced aggregation in washed rabbit platelets (WRPs), which internationally is a well-accepted methodology. PAF along with COX-1, ADP, and PAR-1 (thrombin receptor) constitute major agonists to platelet activation [22]. It exerts its biological action upon binding to its G-protein coupled receptor PAFR (PAF-receptor), which is present on the plasma membrane of cells or intracellular membranes [21].

In particular, and since PAF is a potent lipid mediator of inflammation [23], inhibition of its biological activity (anti-PAF potency) by members of this category, seems to be a very promising strategy for applications in the treatment of inflammatory diseases [14,24].

**Figure 3:**
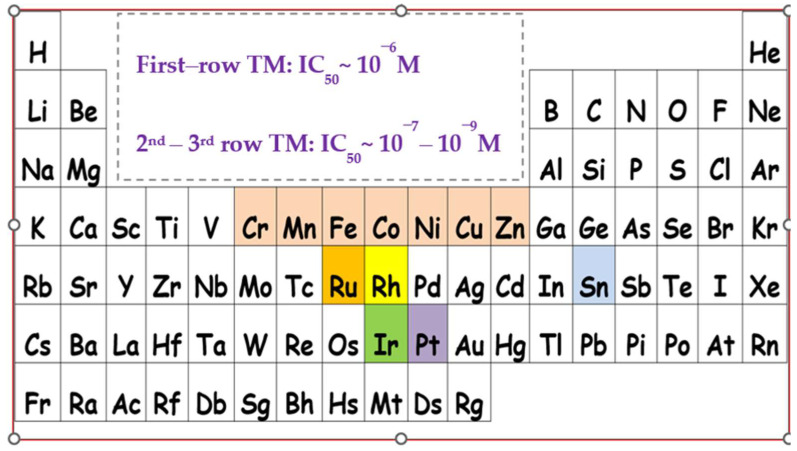
Metal complexes bearing the pqx ligand, that have been evaluated as potent inhibitors of PAF and thrombin-induced aggregation, by our group (TM: transition metal). Colors showcase the different metal ions.

Following the strategy reported above, several coordination compounds and organometallic complexes have been investigated in our group, while preliminary structure-activity relationships have been established [17]. Existing complexes expand from rhodium [25,26,27,28] and iridium [29], to ruthenium [30,31] and tin-based inhibitors, with inhibitory effects of the PAF-induced aggregation in WRPs [32], in the nanomolar and sub-micromolar range. It would be very useful to mention here, that the term “metal-based *inflammatory mediators*” has been recently coined in the literature [33], indicating our contributions in the field. Independently, the antiplatelet activity of some ruthenium(II) complexes has been reported by others, refs. [34,35] highlighting the need for new molecular compounds that could be added to the therapeutic armamentarium (Figure 3).

Undoubtedly, first-row transition metal complexes are particularly attractive candidates compared to purely organic molecules. This, among other reasons, could be attributed to the different coordination numbers and geometries they display, the variable oxidation states obtained, along with the fact that the relevant metal ions participate in several important biological activities that are believed to be essential for mammalian biochemistry [36].

This study has been undertaken to further explore the vast area of metal-based coordination compounds that could be potentially tested as inhibitors against PAF-induced biological activities and probably as anti-inflammatory agents. Thus, we took advantage of the previous knowledge on the rich coordination chemistry of the 2-(2′-pyridyl)quinoxaline ligand (pqx), well-established in our group [37,38] and by others [39,40]. This ligand combines the chelating ability of 2,2′-bipyridine (bpy) [41] with the bridging properties of quinoxaline [42] and belongs to a class of bidentate ligands whose coordination chemistry has been extensively studied [43].

First-row transition metal complexes, from Cr to Zn, incorporating the pxq ligand precursor are interesting examples, that could help us to predict or interpret the biological behavior (antiplatelet activity) of several other well-characterized coordination compounds that display similar structures. The corresponding zinc(II) complex was also tested for comparison, although zinc, a group 12 metal, is not always classified as a transition metal [44].

Herein, we report on the antiplatelet and antithrombotic activities, of a series of known (except the chromium analog) first-row transition metal complexes, incorporating the bidentate ligand 2-(2′-pyridyl)quinoxaline (pqx). The mononuclear *mer*-[Cr(pqx)Cl_3_(H_2_O)] (**1**), [Co(pqx)Cl_2_(DMF)] (**2**) (DMF = *N*,*N*′-dimethyl formamide), [Cu(pqx)Cl_2_(DMSO)] (**3**) (DMSO = dimethyl sulfoxide), [Zn(pqx)Cl_2_] (**4**)) and dinuclear complexes [Mn(pqx)(H_2_O)_2_Cl_2_]_2_ (**5**), [Fe(pqx)Cl_2_]_2_ (**6**) and [Ni(pqx)Cl_2_]_2_ (**7**)) were synthesized and tested against the PAF- and thrombin-induced aggregation, in washed rabbit platelets (WRPs). The antithrombotic potency of these complexes was studied by other well-established thrombotic mediators and platelet agonists, such as thrombin. This is the first time that such an investigation has been performed for first-row transition metal complexes. The biological behavior of the tested compounds on PAF basic metabolic enzymatic pathways was further evaluated along with cell viability assays in HEK293T (human embryonic kidney cells) and HeLa cells (cervical cancer cells), via the 3-(4,5-dimethylthiazol-2-yl)-2,5-diphenyl tetrazolium bromide (MTT) assay.

## 2. Results and Discussion

### 2.1. Chemistry

**Figure 4:**
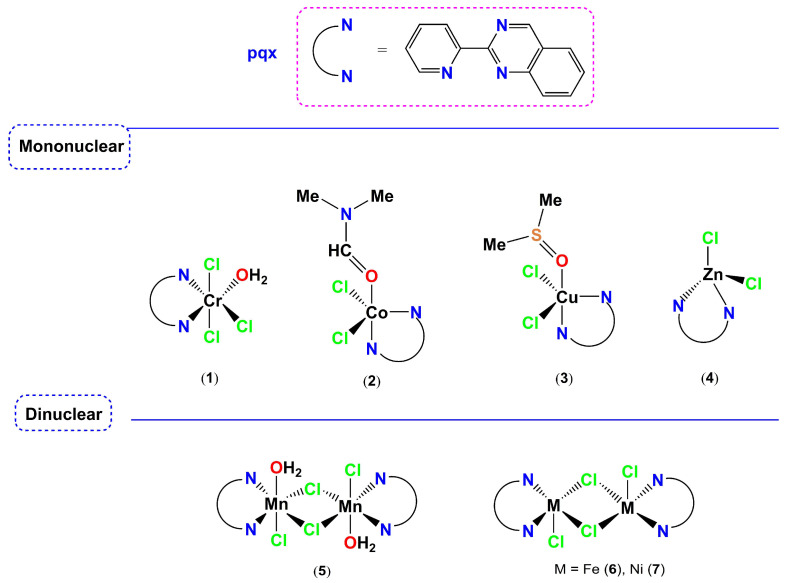
Molecular structures of complexes **1**–**7** described in this study.

The synthesis of all complexes described herein, has been performed according to the literature reports (*vide infra in Materials and Methods*). The yields range from 65–75% (quantitatively for **3**) and their molecular structures are depicted in Figure 4. The synthesis of the iron(II) and nickel(II) dinuclear complexes **6** and **7**, was carried out under aerobic conditions, using common organic solvents, without changes in the yield. In the solid state, complexes **2**, **3**, **6** and **7** show a distorted trigonal bipyramidal geometry, **1** and **5** adopt an octahedral coordination geometry while in complex **4**, zinc resides in a tetrahedral environment, respectively. All complexes are paramagnetic, except for complex **4** which is diamagnetic. NMR data for **4** have been published previously.

The ATR-FTIR spectra of all complexes (**1** and **3** in KBr) are presented in Figures S1–S7 of the Supplementary Materials, including those of **6** and **7** that were not reported previously. From these spectra, it is seen that the pqx ligand has been successfully coordinated to the metal centers affording the relevant complexes. In general, the FT-IR data are in agreement with those of the published procedure (vide infra Section 3). From the FT-IR spectra of **6** and **7**, the presence of water of crystallization, is evident from the intense bands at ~3350–3250 cm^−1^ [45] due to the antisymmetric and symmetric –OH stretching vibration modes. For complex **7,** however, the intense bands reported above are covered by a broad band centered at ~3400 cm^−1^ that may be attributed to the hydrogen-bonded ν(O-H) vibration mode [45]. In addition, the strong band at ~1642 cm^−1^ is due to the δ(H-O-H) bending mode. The FT-IR spectra of both complexes display characteristic ν(C-H) aromatic stretching vibrations at 3096 cm^−1^ (**6**) and 3089 (**7**) cm^−1^, while the intense vibration bands of the pqx ligand dominate in the fingerprint region [43]. In this respect, the characteristic is the sharp single band at 963 cm^−1^ (**6**) and 972 cm^−1^ (**7**), indicating monodentate coordination of quinoxaline [43].

The mononuclear complex [Cu(pqx)Cl_2_(DMSO)] (**3**) was isolated quantitatively, from [Cu(pqx)Cl_2_]_2_ [46], upon treatment with DMSO. Characterization of the DMSO adduct includes UV-Vis and FT-IR spectroscopy, satisfactory elemental analysis, and a single-crystal diffraction study.

The FT-IR (in KBr) spectrum of **3** displays characteristic ν(C-H) aromatic stretching vibrations at 3062 cm^−1^, while the ν(C-H) aliphatic stretching vibrations appear at 2996 and 2908 cm^−1^, respectively. In the region of 1000–400 cm^−1^, the IR spectrum is dominated by the characteristic in-plane and out-of-plane deformation bands of the pyridine ring that are typical for the pqx ligand. These bands are shifted to higher frequencies, at approximately 647 cm^−1^ and 424 cm^−1^ compared to those of the free ligand (pqx) at 620 cm^−1^ and 401 cm^−1^, indicating coordination of pqx to the Cu(II) metal center, through the pyridyl nitrogen atom [42]. The strong band at ~952 cm^−1^ characterizes the monodentate quinoxaline coordination [37]. In addition, the presence of DMSO molecule in complex **3** is confirmed by the rather strong band at 974 cm^−1^. This vibration band can be tentatively assigned to the stretching vibration mode ν(S=O) of *O*-bonded DMSO, while that at 714 cm^−1^ may be due to the stretching vibration mode of ν(C-S) [45]. This is in accord with other copper(II) complexes with *O*-coordinated DMSO [47]. The broad and medium intensity band at 467 cm^−1^ could be attributed to the ν_s_(Cu-O) stretching vibration, verifying the formation of complex **3** [48].

**Figure 5:**
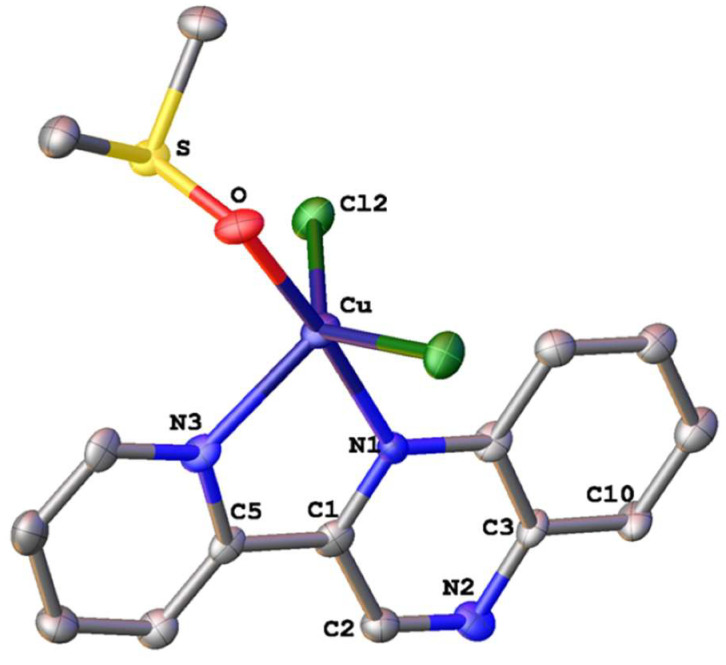
Molecular structure of **3** in the solid state. Thermal ellipsoids are set at 50% probability. Hydrogen atoms are omitted for clarity.

The solid-state structure of **3** was unambiguously determined by single-crystal X-ray diffraction studies. Suitable yellow crystals of **3** were obtained upon the slow evaporation of a DMSO solution of this complex, at ambient temperature, after several days. The molecular structure of the complex is depicted in Figure 5 and selected bond lengths and angles with estimated standard deviations, are included in Tables S1 and S2 (Supplementary Materials).

Complex **3** crystallizes in the monoclinic crystal system and space group *P*2_1_, adopting a trigonal bipyramidal geometry. The equatorial plane is defined by the two chlorine atoms Cl(1) and Cl(2), and the nitrogen atom N(3) of the bidentate ligand (pqx). DMSO ligand coordinated to the metal center via the oxygen atom, occupies the one axial position, while the second axial site is occupied by the N(1) atom of pqx. Distortion from the ideal trigonal bipyramidal geometry is evident by the O-Cu-Cl(1), O-Cu-Cl(2) and O-Cu-N(3) bond angles of 90.45(12)°, 92.00(13)° and 95.40(18)°, respectively. Also from the O-Cu-N(1) bond angle of 174.2(2)°, which deviates slightly from that of 180°. As expected, the Cu-Cl bond distances of 2.324(15) Å and 2.327(16) Å, are slightly shorter as compared to the bridging Cu-Cl bonds (2.473(2) Å) of [Cu(pqx)Cl_2_]_2_ [46,49]. The structural features of **3** differ significantly from those of the CuCl_2_(DMSO)_2_ analog that adopts a distorted square pyramidal geometry, where the Cu-Cl average bond distance is equal to 2.2866(10) Å) [50]. Furthermore, the Cu-O bond distance of 1.970(4) Å, compares well with that reported for similar compounds with *O*-coordinated DMSO [51]. Finally, the chlorine atom Cl(2) displays non-classical intermolecular hydrogen bonding interactions with the H(14C) hydrogen atom of the DMSO ligand, from an adjacent molecule (distance of C(14C)-H(14)**∙∙∙**Cl(2) = 2.818 Å; bond angle = 176.35°) (Figure S8).

#### 2.1.1. Solution Behavior (Conductivity Measurements, UV-Vis Spectroscopy, Solution ATR-IR Measurements and High Resolution Electrospray Ionization Mass Spectrometry (ESI-HRMS))

**Figure 6:**
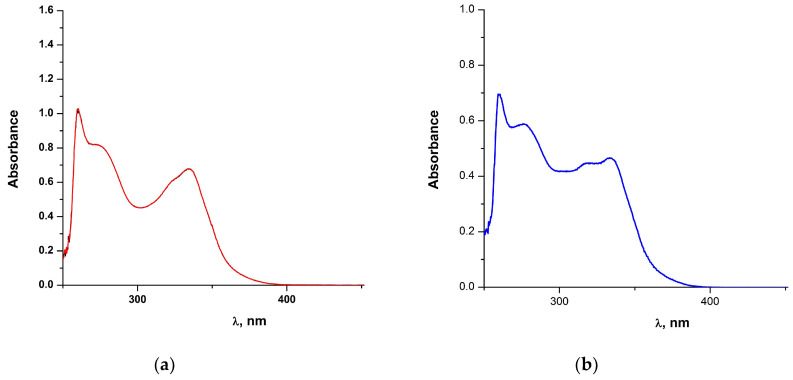
(**a**) The UV-Vis spectrum of **2** in DMSO (7.6 × 10^−4^ M); (**b**) The UV-Vis spectrum of **3** in DMSO (1.7 × 10^−4^ M).

**Figure 7:**
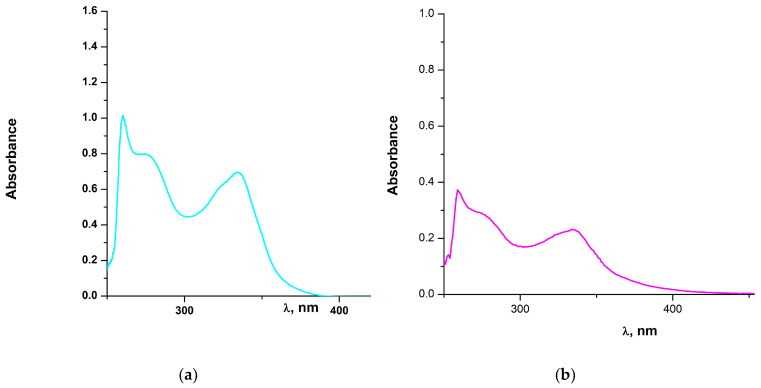
(**a**) The UV-Vis spectrum of **5** in DMSO (2.8 × 10^−4^ M); (**b**) The UV-Vis spectrum of **6** in DMSO (1.5 × 10^−4^ M).

The UV-Vis spectra of all complexes were recorded in DMSO at ambient temperature, immediately after dissolution, as this is the medium used for the biological assays. The UV-Vis spectrum of **1** will be included in a publication that follows. The relevant spectra of complexes **2**, **3**, **5** and **6** are shown below in Figure 6 and Figure 7, respectively, while that of complex **7** (concentrated solution) is included in Figure S9.

The UV-Vis absorption spectra of the light-yellow solutions of complexes **2**, **3**, **5** and **6** were monitored over a period of ~1 to 2 h (not shown) to ensure stability for the relevant biological assays. Notably, the absorption maxima, shape and intensity of the absorption spectra were not modified over time. In addition, it can be clearly seen that the absorption spectra of these complexes are quite similar, suggesting probably similar molecular structures in DMSO solution. The electronic spectra of all complexes display four rather identical absorption bands in the range of 259–334 nm that can be attributed to π → π* and n → π* or charge-transfer transitions. These bands display medium to weak molar extinction coefficients and are in the expected region for similar complexes [52]. For the cobalt(II) complex **2**, the very low intensity and rather broad absorption, centered at ~680 nm can be attributed to d-d transitions [53]. Also for the copper(II) complex **3**, the broad absorption band in the range of 660–870 nm, is attributed to d-d transition [54].

To obtain an additional insight into the solution behavior of complexes **1**, **2**, **5**, **6** and **7** in DMSO, the medium used in the biological assays, in situ IR solution spectroscopy (use of concentrated solutions) was performed. In the mid-IR region, the S=O stretching mode, located at ~1042 cm^−1^, is the dominating absorption band [55]. Thus, upon dissolution of **1** and **5** in DMSO (Figure S10), the formation of a new and very strong peak at 1015 cm^−1^ (**1**) (1018 cm^−1^ (**5**)) becomes evident, next to the central peak of the free solvent at 1042 cm^−1^. This is also the case for complexes **6** and **7**, where the new band, broad and intense, appears at 1018 cm^−1^ (**6**) and 1020 cm^−1^ (**7**), respectively. At first glance, this intense peak could be indicative of the formation of DMSO-adducts in solution. However, we realized that this new peak at ~1018 cm^−1^ disappears upon working with dry DMSO. This observation is in accordance with the literature reports where this peak was attributed to the -S=O bond of hydrogen-bonded DMSO [56]. Thus, we may be very cautious in the interpretation of the new peaks that are observed in this region when DMSO is the medium of choice. Notably, for complexes **1** and **5**, the replacement of the coordinated water molecule occurs, as verified by the presence of a very broad band centered at ~3350 cm^−1^, for the ν(O-H) vibration mode, along with that of the δ(H-O-H) mode at 1631 cm^−1^ (**1**) and 1654 cm^−1^ (**5**) respectively. For the case of the DMF adduct **2** in dry DMSO, we noticed a characteristic peak at 1671 cm^−1^ which can be assigned to free DMF [57]. Apparently, this can be attributed to the rapid displacement of the DMF solvent by DMSO in complex **2,** as proved by ESI-MS spectrometry (vide infra). In addition, the new peak at 994 cm^−1^ is in the expected region for *O*-bonded DMSO. At this point, we may notice that in DMSO (in dilute solutions), the peaks of coordinated pqx ligand suffer from rather low intensity as opposed to those of the ν(S=O) modes that dominate. However, working with concentrated solutions, the weak to medium intensity vibration bands at 418 cm^−1^ and 670 cm^−1^ for (**1**), 419 cm^−1^ and 638 cm^−1^ for (**2**), 419 cm^−1^ and 669 cm^−1^ for (**5**), 419 cm^−1^ and 672 cm^−1^ for (**6**), 423 and 650 cm^−1^ for (**7**)) were assigned to the characteristic deformation bands of the pyridine ring [42]. This is of interest denoting that in DMSO solution (dry or non-dehydrated) the pqx ligand remains coordinated to the metal centers. Further proof is provided from the ATR-IR spectra of the solids recovered after evaporation of DMSO. These are practically identical to each other, as the vibration modes referring to the pqx ligand, appear at approximately the same frequency, with similar patterns and intensities. In addition, the formation of new and intense peaks, due to the S=O vibration modes of *O*-bonded DMSO, is evident. For **2**, the absence of coordinated DMF ligands, and the appearance of coordinated DMSO molecules, is in accordance with the solution IR data reported above.

Molar conductance measurements of the relevant complexes were also performed in DMSO. The *Λ* values of the complexes **1** and **2**, upon dissolution in this medium, were found to be 31 and 49 S cm^2^ mol^−1^, respectively, which is the suggested *Λ* range for a 1:1 electrolyte in DMSO [58,59]. For the dinuclear complexes **5**–**7**, the *Λ* values of 46 S cm^2^ mol^−1^, 30 S cm^2^ mol^−1^ and 40 S cm^2^ mol^−1^, respectively, indicate the ionic character of these complexes suggesting also 1:1 electrolyte. Based on these results we may suggest that in DMSO the chloride bridges between the metal centers weaken, causing the splitting of the dimeric units and subsequent formation of monomeric solvent species. Thus, according to the conductivity values, it can be proposed that one chloro ligand is replaced by DMSO. A second chloro ligand does not undergo exchange by another DMSO ligand, since this would result in a 1:2 type electrolyte, with a significantly higher *Λ* value. Accordingly, subsequent coordination of DMSO may occur to fill the coordination sphere, resulting, therefore, in the conversion of the neutral complex to the uni-univalent ion pair of the possible general type [M(pqx)Cl_χ_(DMSO)_y_]^+^Cl^−^, (M = Mn, Ni: χ = 1, y = 1; M = Fe: χ = 1, y = 3). Possible formulae of the ion pairs formed are given below in the ESI-MS part, while for the case of Fe the χ, y values vary. On the other hand, for **3** and **4**, the *Λ* values of 1–2 S cm^2^ mol^−1^ verify the non-electrolytic behavior of these complexes in DMSO.

To this end, additional proof of the possible nature of the species formed in the DMSO solution is provided by high-resolution electrospray ionization mass spectrometry (ESI-HRMS). For the pqx ligand, the ion at *m*/*z* 208 is typical for protonated [pqx + H]^+^. Molecular ions detected at higher *m*/*z* may be attributed to methanol and DMSO adducts, though the experimental differs from the calculated exact mass (Figure S11). Under the working conditions used, molecular ions corresponding to DMSO adducts are observed except for complex **4**. For the mononuclear complexes **1** and **2**, the ESI-HRMS spectra in the DMSO/methanol mixture present typical peaks at *m*/*z* 484.98 and 378.99, that correspond to the gaseous monocations [Cr(pqx)Cl_2_(DMSO)_2_]^+^ and [Co(pqx)Cl(DMSO)]^+^, respectively (Figures S12 and S13). The formation of these species in DMSO can be described according to Equations (1) and (2).

[Cr(pqx)Cl_3_(H_2_O)] + 2DMSO → [Cr(pqx)Cl_2_(DMSO)_2_]Cl + H_2_O (1)

[Co(pqx)Cl_2_(DMF)] + DMSO → [Co(pqx)Cl(DMSO)]Cl + DMF (2)

For cobalt(II) complex (**2**) the weak intensity ion at *m*/*z* 458.81 can be assigned to the relevant ion [Co(pqx)Cl_3_(DMSO)]^+^. In the case of complex **3,** the typical ions at *m*/*z* 304.98 and *m*/*z* 382.99 can be assigned to the monocations [Cu(pqx)Cl]^+^ and [Cu(pqx)Cl(DMSO)]^+^, respectively (Figure S14). For the diamagnetic complex **4**, the ion at *m*/*z* 305.98 is indicative of the monocation [Zn(pqx)Cl]^+^ (Figure S15). The molecular composition of **5** in the DMSO/methanol mixture was confirmed by electrospray mass spectral data, exhibiting peak envelopes at *m*/*z* 374.99 which correspond to the gaseous monocation [Mn(pqx)Cl(DMSO)]^+^, with the correct isotope pattern (Figure S16). Other weak intensity ions of the formulae [Mn(pqx)Cl]^+^ (*m*/*z* 296.98) and [Mn(pqx)Cl_3_(DMSO) + 6H]^+^ (*m*/*z* 450.81) were detected; isotopic fitting for the latter ion differs slightly from the theoretical one. Taking into consideration, that the gaseous species formed under soft ionization conditions (upon ESI-HRMS) represent the species that exist in the sprayed solution, the ESI-HRMS spectrum confirms the formation of the relevant cation [MnCl(pqx)(DMSO)]^+^ and thus the existence of the equilibrium which is described from Equation (3).

[Mn(pqx)Cl_2_(H_2_O)_2_]_2_ + 2DMSO → 2[Mn(pqx)Cl(DMSO)]Cl + 4H_2_O (3)

For the iron(II) dinuclear complex **6** to the monocations [Fe(pqx)(DMSO)]^+^, [Fe(pqx)(DMSO)_3_]^+^ and [Fe(pqx)Cl(DMSO)_3_]^+^, respectively. The molecular ions detected differ slightly from the calculated exact mass (Figure S17). In the case of the nickel(II) complex **7** the representative peak detected at *m*/*z* 377.99 can be identified as the monocation [Ni(pqx)Cl(DMSO)]^+^, as reported also for the case of the manganese(II) complex **5** (Figure S18). These results corroborate those reported above from molar conductance measurements.

### 2.2. Evaluation of Biological Activity

The biological assays (anti-PAF and antithrombotic studies) of the relevant first-row transition metal complexes **1**–**7,** were carried out in DMSO. Stability in this medium was confirmed by means of UV-Vis spectroscopy, and molar conductance measurements and the identity of the species formed in the solution was proved by ESI-HRMS (Section 2.1.1). The in vitro inhibitory effect of the present compounds on PAF-induced platelet aggregation towards washed rabbit platelets (WRPs) in relation to their diverse structural features (coordination geometry) is reported. The inhibitory potency of these complexes towards thrombin was also investigated, considering the formation of monomeric species in solution, for complexes **5**, **6** and **7,** respectively, as suggested by ESI-HRMS techniques. As far as the biological behavior of the tested compounds on PAF basic metabolic enzymatic pathways is concerned, there are also interesting results which are described below in Section 2.2.3, while cell viability assays are reported in Section 2.2.4.

#### 2.2.1. Inhibitory Effects against the Biological Activities of PAF in Washed Rabbit Platelets (WRPs)

The inhibitory effect of the first-row transition metals bearing the pqx ligand precursor, against the PAF-induced aggregation in WRPs and thrombin is expressed by the IC_50_ values in μM. The IC_50_ values reflect the inhibition strength of each compound since the lowest IC_50_ value reveals the stronger inhibition against PAF-induced aggregation. From the results listed in Table 1, inhibition against the PAF/PAF-R and Thrombin/PAR-3 was in the micromolar and sub-micromolar range. To the best of our knowledge, this is the first time that the antiplatelet (anti-PAF) and antithrombotic properties of first-row transition metal elements with pqx ligands, were tested. Previous studies with this ligand precursor include the heavier atoms of group 8 and group 9, namely ruthenium, and rhodium, respectively. Thus, the square-planar rhodium(I) complex [Rh(cod)(pqx)]Cl (cod = *cis*-1,5-cyclooctadiene; IC_50_ = 15 nM), the octahedral rhodium(III) complex *mer*-[Rh(pqx)Cl_3_(MeOH)] (IC_50_ = 0.125 μM), along with the ruthenium(II) analog [Ru(dcbpyH_2_)_2_ (pqx))](NO_3_)_2_ (dcbpyH_2_ = 2,2-Bipyridine-4,4′-dicarboxylic acid; IC_50_ = 0.18 μM) exhibit inhibitory effects against the PAF-induced aggregation in the nano and sub-micromolar range [17,25].

In WRPs, the metal precursors studied, exhibited diverse inhibitory effects. In fact, CrCl_3_ × 6H_2_O, MnCl_2_ × 4H_2_O and FeCl_2_ × 4H_2_O showed weaker inhibitory effects, with IC_50_ values ranging from 2.6 to 5.6 mM, while CoCl_2_ × 6H_2_O showed negligible effects. On the other hand, NiCl_2_ × 6H_2_O (IC_50_ = 30 μM), CuCl_2_ × 2H_2_O (IC_50_ = 22 μM and ZnCl_2_ (IC_50_ = 30 μM) were more active, by almost an order of magnitude. Their potency is in the same range as that of pqx (IC_50_ = 32 μM) and their IC_50_ values were significantly lower than those of the Cr(III), Mn(II) and Fe(II) metal precursors.

From the results shown in Table 1, we may also indicate that upon coordination of pxq to Cr(III) and Fe(II) ions affording complexes **1** (IC_50_ = 4.5 μM) and **6** (IC_50_ = 1.79 μM), respectively, a pronounced two-orders of magnitude drop in the IC_50_ value is observed (inhibitory effect increases). This trend is also followed for the case of Ni(II), Mn(II), and Cu(II) ions, as clearly expressed by a four-fold decrease in the IC_50_ value for Ni(II) (IC_50_ = 6.83 μM), an almost 10-fold decrease for Mn(II) (IC_50_ = 39 μM) and a two-fold decrease for Cu(II) (IC_50_ = 10.6 μM) as well. Notably, upon coordination of Zn(II) ions to pqx, the IC_50_ value is decreased by approximately an order of magnitude (IC_50_ = 3.3 μM). These findings are in accord with the well-established general trend known as the *synergetic effect*, indicating that coordination of the ligand precursor to a metal center, results in an increase in the antiplatelet activity of the relevant compound [28]. As recently demonstrated [32], this trend can be clearly attributed to the overall structure of the obtained complexes, signifying that an efficient combination of the ligand and metal precursors, providing the target molecule, is the key component for increased activity against the PAFR.

For WRPs, the results of Table 1, further indicate that within the complexes tested, the Fe(II) complex **6**, is the most potent inhibitor against the PAF-induced aggregation (IC_50_ = 1.79 μM). All other complexes, including the six-coordinate Cr(III) complex **1**, exhibit an anti-PAF activity in the micromolar range (IC_50_ from ~4 μM to ~11 μM). Notably, the tetrahedral complex [Zn(pqx)Cl_2_] **4**, is quite potent, as compared to **6**; exhibiting a two-fold decrease in the PAF inhibitory effect, while the Mn(II) complex **5** was the less active (IC_50_ = 39 μM). The results reported above clearly demonstrate the tremendous impact of the nature of metal ions on the biological potency exerted by the metal complexes studied. In other words, Fe(II), Zn(II), Co(II), Ni(II), Cr(III) and Cu(II) ions, seem to be important for the PAFR activity of the relevant complexes incorporating the pqx ligand precursor (Figure 3).

Complementary, the size of the target molecule (relevant metal-based inhibitors) seems to affect, the biological activity expressed. Our findings based on theoretical docking calculations propose that the less bulky inhibitor that fits well within the binding site of the PAF receptor is more potent as opposed to the bulkier one, which could bind to the extracellular domain of the receptor, antagonizing the substrate’s entrance to PAFR [25,32]. Accordingly, within the series of the complexes studied, we can propose that the Mn(II) complex **5** is the bulkiest, rendering it less effective against the PAF-induced aggregation (IC_50_ = 39 μM), a result which is in favor of the preliminary structure-activity relationship established [32]. This also corroborates with previously reported results [17,25], as for example the square-planar rhodium(I) complex [Rh(cod)(pqx)]Cl (less bulky) that exhibits a considerably increased anti-PAF activity (IC_50_ = 15 nM), compared to the bulkier, octahedral rhodium(III) complex *mer*-[Rh(pqx)Cl_3_(MeOH)] (IC_50_ = 0.125 μM)].

A direct comparison of the anti-PAF activities of our complexes with analogous ones, displaying similar ligand precursors around the metal periphery, cannot be made in a straightforward manner, since there are no reports on such systems. However precious information could be gathered, from a series of metal complexes (M = Cu, Co, Ni, Zn) with chalcogenated imidodiphosphinato ligands. These complexes display an inhibitory effect towards the PAF-induced aggregation, which is in the same range as for the complexes described herein [17,60]. Also, additional information is provided from a series of N-heterocyclic carbene (NHC) aurates, though this research was performed with hPRPs [61]. By comparing the anti-PAF inhibitory potencies of complexes **1**–**7**, we noticed that these are similar to those reported for a series of Rh(III) (IC_50_ = 0.1–2.6 μM) [26], Ir(III) [8 μM] [29] as well as for Ru(II) and Ru(III) (IC_50_ = 0.2–7 μM) PAFR antagonists, [17,30].

Interestingly, the biological activity of the metal complexes presented herein, against the PAF-induced aggregation in WRPs, is comparable with the inhibitory effect of known natural PAF antagonists from the series of Gingolides B, namely BN 52020 (IC_50_ = 3.6 μM), BN 52021 (IC_50_ = 9.7 μM) along with that of rupatadine, which exhibits an IC_50_ value of 0.25 μM, respectively.

Conclusively, from the experimental findings reported above, the inhibitory activity of the complexes tested drops in the following order: **6** > **4** > **2** > **1** > **7** > **3** > **5**.

#### 2.2.2. Inhibitory Effect against Thrombin

Considering the interesting results against the PAF-induced aggregation in WRPs we further examined the antithrombotic activity of the ligand and metal precursors, along with that of metal complexes **1**–**7**. From the results collected in Table 1, important features arise, when comparing them. Thus, ongoing from CrCl_3_ × 6H_2_O to **1**, the antithrombotic activity drops almost eleven times (from 611 μM to 54.6 μM), while in the case of MnCl_2_ × 4H_2_O the inhibitory activity is reduced almost 33 times (from 466 μM to 14 μM). Similar results are found for the zinc analog **4**, where a ~12-fold decrease in its inhibitory potency against thrombin-induced aggregation is observed. These results clearly demonstrate that the *synergetic effect*, is the relative activity trend that is followed for the case of thrombin as well. Notably, iron(II) complex **6** showed the strongest antithrombotic activity with an IC_50_ value of 0.46 μM, rendering it the best compound in this series. For complexes **3** (IC_50_ = 3.1 μM) and **7** (IC_50_ = 5.60 μM), however, the inhibitory activity is slightly reduced in comparison to that of the metal precursors CuCl_2_ × 2H_2_O and NiCl_2_ × 6H_2_O, respectively. In any case, both complexes (**3** and **7**) exhibit the highest inhibition in comparison to that of pqx, which is not active toward thrombin-induced aggregation. This is a remarkable feature that highlights the tremendous impact of the relevant metal ion within the overall structure of the metal complex. In fact, *cis*-[Ru(dcbpyH_2_)_2_(pqx)](NO_3_)_2_ (dcbpyH_2_ = 2,2′-Bipyridine-4,4′-dicarboxylic acid), a known Ru(II)-pqx complex is inactive against thrombin while the Fe-pqx complex (**6**), is the most potent in the series [17]. This could be indicative of the presence of Fe(II) in complex **6**, which is a lighter analog within group 8 metals. At this point, it is interesting to mention that the antithrombotic potency of the iron(II) complex **6** is comparable to that of the recently reported organotin(IV) analogs L_OEt_SnPh_3_ (IC_50_ = 0.6 μM) and L*_OEt_SnPh_3_ (IC_50_ = 0.23 μM), where L_OEt_ and L*_OEt_ stand for the oxygen tripodal ligand [(*η*^5^-C_5_R_5_)Co{P(OEt)_2_O}_3_]^−^, {R = H (L_OEt_^−^), Me ((L*_OEt_^−^); Et = -C_2_H_5_). Accordingly this potency is in the same range to that reported for a series of Ru(II) thrombin inhibitors, namely *cis*-[Ru(bpy)_2_(dcbpyH_2_)](PF_6_)_2_ (bpy = 2,2′-bipyridine; IC_50_ = 4.5 μM) and *cis*-[Ru(dcbpyH_2_)_2_(L)](NO_3_)_2_ (L = 4-carboxy-2-(2′-pyridyl)quinoline; IC_50_ = 6.1 μM) [30]. To this end, we may suggest that the Cr(III) complex (**1**), exhibits higher selectivity towards PAF aggregation since inhibition against thrombin was significantly lower (twelve-fold decrease in the IC_50_ value). As a result, the inhibitory activity of the complexes tested, against thrombin-induced aggregation decreases in the following order: **6** > **3** > **4** > **7** > **2** > **5** > **1**.

Based on these preliminary results, we may notice that the Fe-pqx complex (**6**), is the most potent inhibitor, exerting similar affinities for both the PAFR and PAR receptors while the Cr-pqx complex (**1**) proved to be the less effective against thrombin induced aggregation. It is also to be mentioned that the inhibitory activity of the Mn-pqx analog (**5**) drops dramatically in both cases (PAFR and PAR receptors), rendering **5** less potent.

#### 2.2.3. Biosynthesis of PAF

**Figure 8:**
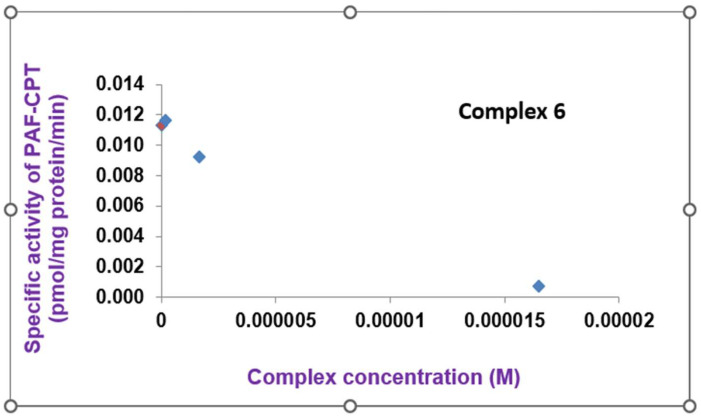
The inhibition of the specific activity of dithiothreitol-insensitive PAF choline phosphotransferase (PAF-CPT) by the Fe(II) complex **6**. The orange bullet represents the blank solution while the blue bullets display the reductive trend of the Fe(II) complex **6** against enzyme activity.

The Fe(II) complex **6** was found to inhibit, in a dose-dependent way, the specific activity of dithiothreitol-insensitive PAF choline phosphotransferase (PAF-CPT), one of the main biosynthetic PAF enzymes responsible for the maintenance of PAF levels in the organism, but also connected to chronic inflammatory processes (Figure 8).

**Figure 9:**
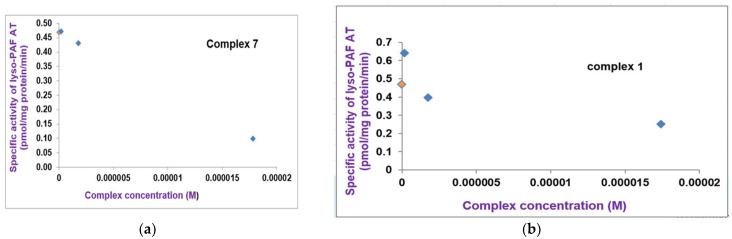
(**a**) The inhibition of the specific activity of lyso-PAF:acetyl-CoA acetyltransferase (lyso-PAF-AT), by the Ni(II)-pqx (**7**) complex. The green bullet represents the blank solution while the blue bullets display the reductive trend of the Ni(II)-pqx (**7**) complex against enzyme activity; (**b**) The inhibition of the specific activity of lyso-PAF:acetyl-CoA acetyltransferase (lyso-PAF-AT), by the Cr(III)-pqx (**1**) complex. The green bullet represents the blank solution while the blue bullets display the reductive trend of the Cr(III)-pqx (**1**) complex against enzyme activity.

In addition, the Ni(II)-pqx (**7**) and Cr(III)-pqx (**1**) complexes were found able to inhibit in a dose-dependent way the specific activity of lyso-PAF:acetyl-CoA acetyltransferase (lyso-PAF-AT), the other main biosynthetic PAF enzyme, linked to acute inflammatory processes (Figure 9).

**Figure 10:**
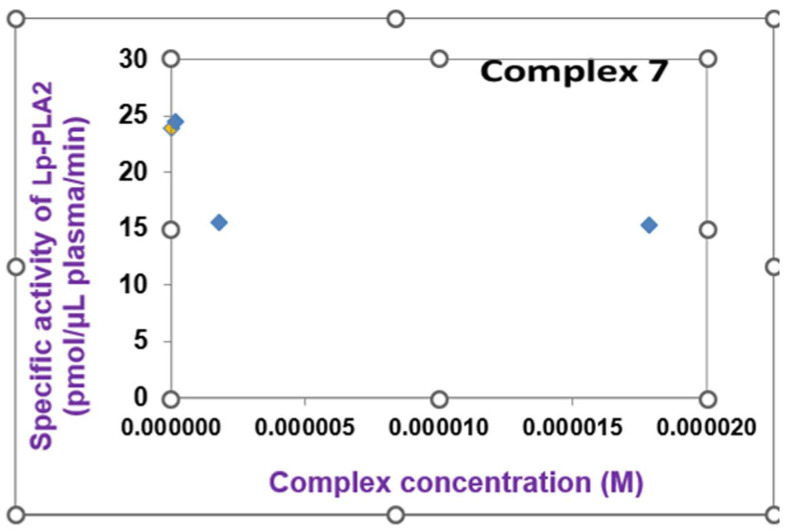
The inhibition of the specific activity of Lipoprotein-associated phospholipase A2 (Lp-PLA2), by the Ni(II)-pqx (**7**) complex. The yellow bullet represents the blank solution while the blue bullets display the reductive trend of the Ni(II)-pqx (**7**) complex against enzyme activity.

However, in increased concentrations, the Ni(II)-pqx (**7**) complex disables the enzymatic activity of PAF catabolism in plasma moderated by Lipoprotein-associated phospholipase A2 (Lp-PLA2) as shown in Figure 10.

Taking into consideration that PAF is a key mediator of the inflammatory processes in numerous pathological conditions, the ability of the Cr-pqx (**1**), Fe-pqx (**6**) and Ni-pqx (**7**) complexes to (i) affect in vitro PAF aggregation by interacting with PAF receptor and (ii) affect key biosynthetic and catabolic enzymes comes in accordance with several previous experiments, providing a new perspective in anti-inflammatory properties of the complexes under investigation and their potential pharmaceutical use. However, in vivo studies must follow in order to confirm the aforementioned in vitro conclusions.

#### 2.2.4. Cell Viability Assay

In this report, cell viability tests were conducted in HEK293T and HeLa cancer cells by means of the colorimetric 3-(4,5-dimethylthiazol-2-yl)-2,5-diphenyl tetrazolium bromide (MTT) assay after exposure for 48 h. A HeLa cancer cell line was selected since it is widely used in cytotoxicity studies [62]. For all complexes, a 10 mM stock solution in DMSO (H_2_O for cisplatin) was prepared and diluted appropriately to reach the final concentration reported in the Materials and Methods. The non-cancerous HEK293T cell line was used for comparison. The IC_50_ values of the first-row metal complexes, the pqx ligand and cisplatin, presented herein were calculated using a dose-response model, which was obtained from sigmoidal fitting of dose-response curves [63]. Cytotoxicity results of all complexes and pqx ligands in both cell lines, expressed as IC_50_ mean values, are summarized in Table 2.

From the results listed herein (Table 2), it becomes evident that in the HEK293T cell line, the Cu-pqx complex (**3**) was the most cytotoxic with an IC_50_ value of 2.10 ± 0.52 exhibiting even higher cytotoxicity than cisplatin. All other complexes were less toxic than cisplatin. Accordingly, cytotoxicity in HEK293T cells decreases in the following order: **3** > cisplatin > **5** ≅ **7** > **6** > **2** > **4** ≅ pqx > **1** suggesting that pqx, Cr-pqx (**1**) and Zn-pqx (**4**) compounds show negligible cytotoxic effects in this cell line compared with the other substances included in Table 2. Even though cisplatin was toxic to both cell lines, its effects were more potent in the HEK293T cell line.

Our results indicated a variability in the cytotoxicity action of the complexes **1**–**7** in tumor-derived HeLa cells, which in some cases was even lower than the cytotoxicity observed in HEK293T cells. The cytotoxicity profile of the tested compounds drops in the following order: cisplatin > **3** > pqx > **6** > **4** > **1** ≅ **7** > **2** > **5**. The most toxic compound in HeLa cells was complex **3** displaying almost double IC_50_ values compared to cisplatin. However, since complex **3** also showed a dramatic decrease in the viability of HEK293T cells, a non-cancerous immortalized cell line, it cannot serve as a potential metallodrug for cancer therapy. Notably, the cytotoxicity of the pqx ligand precursor in this cell line is significantly higher compared to the cytotoxicity reported for the mononuclear (**1**, **2**, **4**) and the dinuclear complexes (**5**–**7**), respectively. In addition, the Cr-pqx (**1**), Co-pqx (**2**) and Ni-pqx (**7**) complexes, only slightly affected the HeLa cancer cells whereas the Mn-pqx complex **5** was practically non-cytotoxic within the various concentrations tested, rendering it the less potent for this cell line. On the other hand, the Zn-pqx complex (**4**), and to a lesser extent the Fe-pqx complex (**6**) may be considered possible candidates for cancer therapy, since their potencies (IC_50_ values) fall within the same range or are lower compared to those reported for the HEK293T line. The observed cytotoxic activity of the complexes reported herein is comparable to that of other metallodrugs carrying similar bidentate ligands, i.e., [Co_2_(L)_2_(bipy)_2_]_n_ (where bipy = 2,2′-bipyridine, and L = biphenyl-2,4′-dicarboxylic acid) with IC_50_ = 17.61 ± 3.58 [64]. Notably, the pqx metal complexes are also more effective than the cytotoxicity reported for the [(η^6^-p-cymene)RuCl_2_L] (L = 4-cyanopyridine (I), 4-aminophenol (III), pyridazine (IV), and [(η^6^-p-cymene)RuClL_2_]PF_6_; L = cyanopyridine (V), complexes [64].

The obtained cell viability results, further reveal that complex **3**, a potent anti-PAF inhibitor (IC_50_ = 10.6 μM), is more cytotoxic, in HEK293T cells, compared to cisplatin with an IC_50_ value of 0.55 μM against PAF [25]. In line with that, the Fe-pqx complex (**6**) which is a potent sub-micromolar PAF-induced aggregation inhibitor in WRPs (IC_50_ = 1.79 μM), was proved also to be quite effective against the present cell line. This is also the case for the HeLa cancer cells. In this respect, recent reports in the field demonstrate that the administration of PAFR antagonists, such as the WEB2086 one, in combination with chemotherapy, may represent a promising strategy for cancer treatment [65]. Practically this means that the in vivo administration of PAF receptor antagonists may reduce the formation of new vessels in cancer cells [66].

To this end, we may propose that the most potent metal-based *inflammatory mediators* **6** and **4** could be considered as possible antiplatelet, antithrombotic, and anticancer agents. This becomes apparent, since both complexes, exhibit moderate cytotoxicity in HeLa cancer cells and at the same time are highly potent PAF inhibitors (anti-inflammatory activity). This is an interesting approach that considers the positive effects acquired with the administration of a substance that exerts dual anti-PAF and cytotoxic activity. Recent reports highlight that this strategy may inhibit tumor development, decreasing cell proliferation, as already reported in vivo, after animal treatment with a combination of cisplatin and a PAF inhibitor [67]. The results reported in this manuscript clearly demonstrate that metal-based *inflammatory mediators* may be considered potent metallodrugs against the compact of cancer (cancer therapy-induced inflammation) [65]. In any case, further experiments are required to gain an insight on the pharmacological profile of the mediators reported. This would enable us to continue further and check possible entry to clinical trials. Towards this goal, one may suggest that toxicity of metal complexes is a drawback, for further studies. We may comment against that, since for example the Cr-pqx (**1**) and Zn-pqx (**4**) metal complexes proved almost inactive (non-toxic) in healthy cells, (human lymphocytes cultures) followed by the Fe-pqx (**6**) and Co-pqx (**2**) analogs, while their anti-PAF activity was in the micro-molar range (IC_50_ = 1.79–4.5 μM). Obviously, these metal complexes may be considered further for in vivo investigation.

**Figure 11:**
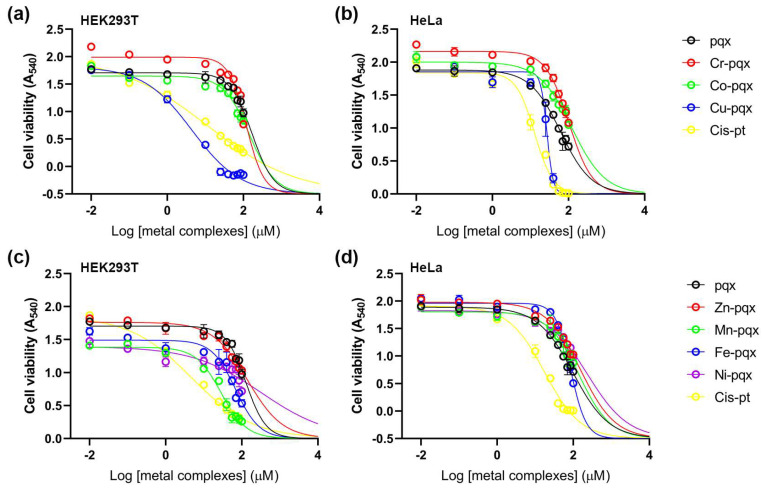
Viability curves for HEK293T (**a**,**c**) and HeLa (**b**,**d**) cell lines for pqx metal complexes.

Data are presented in Figure 11, including cisplatin that served as a positive control.

## 3. Materials and Methods

The ligand 2-(2′-pyridyl)quinoxaline (pqx) was prepared as described in the literature [43]. The complexes examined in this study were prepared according to published procedures: Co(pqx)Cl_2_(DMF) (**2**) [68], [Cu(pqx)Cl_2_]_2_ (**3**) [46], Zn(pqx)Cl_2_ (**4**) [69], [Mn(pqx)(H_2_O)_2_Cl_2_]_2_ (**5**) [70], [Fe(pqx)Cl_2_]_2_ (**6**) [71], [Ni(pqx)Cl_2_]_2_ (**7**) [71]. The detailed synthesis and characterization of the *mer*-[Cr(pqx)Cl_3_(H_2_O)] complex (**1**) will be described elsewhere [72].

All other reagents and solvents were used as such. FT-IR spectra were recorded on a Shimadzu IR Affinity-1 spectrometer as potassium bromide pellets and in ATR mode, in the spectral range of 4000–400 cm^−1^. For the mid-IR spectral range, 16 scans with a nominal resolution of 1 cm^–1^ were averaged. Elemental analyses were obtained from the Microanalysis Center of the Institut für Anorganische Chemie Universität Bonn. UV–Vis spectra were determined in a CARY 17D spectrophotometer, in DMSO. Molar conductance measurements were performed in a WTW-LF3 conductivemeter. The reproducibility of the measurements was examined by repeating each experiment two times, while all data were corrected with the specific conductivity of the solvent. The correction was made by subtracting the specific conductivity of the solvent medium from those of the solutions. The molar conductivities (*Λ*/S cm^2^ mol^−1^) were calculated from the experimental specific conductivities (*k*/S cm^1^) and the concentrations (c/mol L) of the solutions using the equation *Λ* = 10^3^ *k*/c.

For the electrospray ionization mass spectrometry (ESI-MS) experiments, individual solutions of 5 mg L^−1^ were prepared in Methanol LC-MS grade/DMSO 50/50 *v*/*v* and infused directly into a hybrid Quadrupole Time-of-Flight (QToF) mass spectrometer (Maxis Impact, Bruker Daltonics, Bremen, Germany), under a constant flow of 180 μL min^−1^.

### 3.1. Synthesis and Characterization

#### 3.1.1. Synthesis of Complex 3

This complex was prepared from the [Cu(pqx)Cl_2_]_2_ precursor (50 mg, 0.08 mmol), upon dissolution in DMSO (5 mL). The solution’s color changed to brown-yellow, and the mixture was stirred at ambient temperature for about 1 h, to ensure dissolution. The obtained solution was left to evaporate slowly, at ambient temperature, over a period of several days. During this time some X-ray quality crystals were collected. The obtained slurry (and some crystalline solid) was solidified upon repeated treatment with a mixture of acetone/diethy ether (1:10, *v*/*v*) affording **3**, as a light brown-yellow powder that was dried in vacuo at ambient temperature. Yield: quantitative. m.p. 160 °C (changed to brown). (Anal. Calcd for C_15_H_15_Cl_2_CuN_3_OS: C, 42.91; H, 3.60; N, 10.01. Found: C, 42.78; H, 3.54; N, 9.94%. IR (KBr): ν [cm^−1^] = 3062(w) [ν(C-H)_arom_)], 2996(m) [ν(C-H)_aliph_], 2908(m) [ν(C-H)_aliph_], 1597(s), 1503(vs), 1479(vs), 1439(m), 1424(m), 1371(s), 1331(s), 1267(m), 1210(vs), 1156(m), 1132(s), 1112(s), 1079 (s), 1056(s), 986(vs), 974 (vs) [ν(S=O)], 952 (s), 920(s), 803(vs), 785(vs), 772(vs), 741(s), 714(m) [ν(C-S))], 647(s), 573(m), 552(m), 467([ν_s_(Cu-O)_arom_)], 424(w). UV-Vis (DMSO, λ_max_, nm): 332 (ε = 2710 M^−1^ cm^−1^), 317 (ε = 2650 M^−1^ cm^−1^), 276 (ε = 3470 M^−1^ cm^−1^), 260 (ε = 4060 M^−1^ cm^−1^). Λ(DMSO): 1 S cm^2^ mol^−1^. ESI-HRMS (DMSO/MeOH, positive mode): *m*/*z* 304.9781 detected for [C_13_H_9_ClCuN_3_]^+^ (calc. 304.9775), *m*/*z* 382.9927 detected for [C_15_H_15_ClCuN_3_OS]^+^ (calc. 382.9915).

#### 3.1.2. Data for Complexes **1**, **2**, **3**, **4**, **5**, **6** and **7**

Ligand **pqx**: ESI-HRMS (DMSO/MeOH, positive mode): *m*/*z* 208.0873 detected for [C_13_H_10_N_3_]^+^ (calc. 208.0869).

Complex **1**: IR (KBr): ν [cm^−1^] = 3384 (br, s), [n(O-H)], 3128 (w), [ν(C-H)_arom_)], 3066 (w), [ν(C-H)_aliph_)], 1631 (br, s), [d(H-O-H)], 1603(s), 1540(m), 1510(m), 1487(s), 1466(m), 1435(vs), 1369(m), 1342(s), 1265(m), 1219(s), 1152(s), 1125(s), 1080(s), 1060(s), 1038(m), 979(s), 919(m), 803(s), 781(s), 771(s), 749(m), 718(w), 657(m), 576(w), 550(w), 434(m). UV-Vis (DMSO, λ_max_, nm, concentrated solution): 334, 324, 275, 260. Λ(DMSO, 1.1 × 10^−3^ M): 31 S cm^2^ mol^−1^. ESI-HRMS (DMSO/MeOH, positive mode): *m*/*z* 484.9841 detected for [C_17_H_21_Cl_2_CrN_3_O_2_S_2_]^+^ (calc. 484.9852), *m*/*z* 406.9705 detected for [C_15_H_15_Cl_2_CrN_3_OS]^+^ (calc. 406.9718, base peak).

Complex **2**: m.p. > 156 °C (dec). IR (KBr): ν [cm^−1^] = 3062(w), [ν(C-H)_arom_)], 2934(w), [ν(C-H)_aliph_)], 1638(vs), [ν(CO)], 1600(s), 1576(m), 1542(s), 1480(vs), 1430(s), 1371(vs) [[d(*NCH*), 1331(s), 1211(s), 1108(s), 1075(m), 1054(m), 1025(m), 969(s), 901(m), 803(s), 775(vs), 743(s), 572(m), 550(m), 424(s), 404(s)]. UV-Vis (DMSO, λ_max_, nm): 334 (ε = 1800 M^−1^ cm^−1^), 322 (ε = 1620 M^−1^ cm^−1^), 274 (ε = 2180 M^−1^ cm^−1^), 260 (ε = 2680 M^−1^ cm^−1^). Λ(DMSO, 5.0 × 10^−4^ M): 49 S cm^2^ mol^−1^. ESI-HRMS (DMSO/MeOH, positive mode): *m*/*z* 378.9941 detected for [C_15_H_15_ClCoN_3_OS]^+^ (calc. 378.9956, base peak).

Complex **4**: m.p. 190 °C. IR (ATR): ν [cm^−1^] = 3066(w), [ν(C-H)_arom_)], 1601(s), 1546(m), 1505(m), 1482(vs), 1443(s), 1375(m), 1325(s), 1216(s), 1120(s), 1079(m), 1056(m), 1027(m), 976(s), 910(m), 802(s), 779(vs), 770(vs), 743(s), 573(m), 550(m), 417(m), 405(m)]. UV-Vis (DMSO, λ_max_, nm, concentrated solution): 334, 324, 275, 260. Λ(DMSO, 6.1 × 10^−4^ M): 2 S cm^2^ mol^−1^. ESI-HRMS (DMSO/MeOH, positive mode): *m*/*z* 305.9792 detected for [C_13_H_9_ClZnN_3_]^+^ (calc. 305.9771).

Complex **5**: m.p. > 228 °C (dec). IR (ATR): ν [cm^−1^] = 3325(sh, s), [ν(O-H)], 3245 (sh, s), [ν(O-H)], 3075(w), [ν(C-H)_arom_)], 1654(s), 1594(s), 1541(s), 1510(m), 1485(vs), 1434(s), 1374(m), 1328(s), 1213(s), 1112(s), 1078(m), 1059(m), 1011(m), 971(s), 928(m), 802(s), 782(vs), 764(s), 744(s), 573(m), 550(m), 417(m), 405(m)]. UV-Vis (DMSO, λ_max_, nm): 334 (ε = 1800 M^−1^ cm^−1^), 322 (ε = 1620 M^−1^ cm^−1^), 274 (ε = 2180 M^−1^ cm^−1^), 260 (ε = 2680 M^−1^ cm^−1^). Λ(DMSO, 6.0 × 10^−4^ M): 46 S cm^2^ mol^−1^. ESI-HRMS (DMSO/MeOH, positive mode): *m*/*z* 374.9979 detected for [C_15_H_15_ClMnN_3_OS]^+^ (calc. 374.9994).

Complex **6**: m.p. 158 °C. IR (ATR): ν [cm^−1^] = 3096(w), [ν(C-H)_arom_)], 1641(s), 1601(s), 1530(m), 1500(m), 1449(m), 1370(m), 1300(m), 1213(m), 1165(m), 1131(m), 1067(m), 1000(w), 963(s), 885(s), 799(s), 779(vs), 734(m), 547(m), 418(m), 405(m)]. UV-Vis (DMSO, λ_max_, nm): 334 (ε = 1550 M^−1^ cm^−1^), 325 (ε = 1470 M^−1^ cm^−1^), 273 (ε = 1970 M^−1^ cm^−1^), 259 (ε = 2520 M^−1^ cm^−1^). Λ(DMSO, 1.2 × 10^−3^ M): 30 S cm^2^ mol^−1^. ESI-HRMS (DMSO/MeOH, positive mode): *m*/*z* 341.9092 detected for [C_15_H_16_FeN_3_OS]^+^ (calc. 342.0363), *m*/*z* 498.1844 detected for [C_19_H_28_N_3_FeS_3_O_3_]^+^ (calc. 498.0642), *m*/*z* 533.1948 detected for [C_19_H_28_N_3_ClFeS_3_O_3_]^+^ (calc. 533.0331).

Complex **7**: m.p. > 226 °C (dec). IR (ATR): ν [cm^−1^] = 3334(sh, s), [ν(O-H)], 3089(w), [ν(C-H)_arom_)], 1645 (s), 1486(s), 1432(m), 1371(s), 1336(s), 1212(s), 1116(s), 1082(s), 1061(m), 972(m), 920(m), 802(s), 779(vs), 760(m), 744(s), 717(m), 571(m), 547(m), 424(m), 405(m). UV-Vis (DMSO, λ_max_, nm, concentrated solution 1.2 × 10^−4^ M): 334, 325, 273, 259. Λ(DMSO, 2.3 × 10^−3^ M): 40 S cm^2^ mol^−1^. ESI-HRMS (DMSO/MeOH, positive mode): *m*/*z* 377.9972 detected for [C_15_H_15_ClN_3_OS_2_Ni]^+^ (calc. 377.9972).

### 3.2. Single-Crystal X-ray Structural Determination

The X-ray structure analysis was performed on a Nonius KappaCCD diffractometer diffractometer using graphite monochromated Mo-Kα radiation (λ = 0.71073 Å). Ψ-scan was carried out for (extinction coefficient = 0.0016(3)). The crystal was kept at 123.15 K during data collection.

Structure solution was performed with Direct Methods (SHELXS-97, SHELXS-86) and subsequent Fourier-difference synthesis (SHELXL-93, SHELXL-97) without restraints [73]. Refinement on F^2^ was carried out by full-matrix least squares techniques. Non-hydrogen atoms were refined anisotropically. Using Olex2 [74], the structure was solved with the XS structure solution program using the Patterson Method and refined with the XL refinement package using Least Squares minimization.

A summary of the crystal data, data collection and refinement for the structure of **3** is given in Table 3. CCDC 2237537 (**3**), contains the supplementary crystallographic data for this paper. These data can be obtained free of charge from the Cambridge Crystallographic Data Centre via www.ccdc.cam.ac.uk/data_reqeust/cif (accessed on 23 January 2023).

### 3.3. Biological Evaluation

#### 3.3.1. Materials and Methods for Biological Experiments in Washed Rabbit Platelets (WRPs)

The biological experiments of PAF-induced platelet aggregation as well as the evaluation of the inhibitory effect of its synthetic compounds were performed according to thoroughly described protocols [25]. The inhibitory effect of the starting materials (pqx = 2-(2′-pyridyl)quinoxaline, CrCl_3_ × 3H_2_O, MnCl_2_ × 4H_2_O, FeCl_2_ × 4H_2_O, CoCl_2_ × 6H_2_O, NiCl_2_ × 6H_2_O, CuCl_2_ × 2H_2_O, ZnCl_2_) and of the coordination compounds **1**–**7**, towards PAF and thrombin related biological activities (aggregation experiments) in washed rabbit platelets (WRPs) was carried out according to previously described protocols [25]. Briefly, in order to obtain the washed rabbit platelets (WRPs), platelet-rich plasma was washed on Ficoll–Paque cushions and adjusted to 2.5 × 10^−8^ platelets/mL of Tyrode’s buffer, pH 7.2. The dilution of PAF was prepared using pyrogen-free 0.15 M NaCl containing 2.5 mg/mL of crystallized bovine serum albumin, required for dispersion of PAF. The compounds under investigation were firstly dissolved in the appropriate solvent that is: DMSO for pqx (0.010 M), H_2_O for CrCl_3_ × 3H_2_O (0.016 M), H_2_O for MnCl_2_ × 4H_2_O (0.004 M), DMSO-EtOH (4/1, *v*/*v*)) for FeCl_2_ × 4H_2_O (0.014 M), H_2_O for CoCl_2_ × 6H_2_O (0.010 M), H_2_O for NiCl_2_ × 6H_2_O (0.010 M), H_2_O for CuCl_2_ × 2H_2_O (0.008 M), H_2_O for ZnCl_2_ (0.013 M), DMSO for **1** (0.017 M), DMSO for **2** (0.010 M0, DMSO for **3** (0.004 M), DMSO for **4** (0.002 M), DMSO for **5** (0.003 M), DMSO-EtOH-H_2_O (25/1/1, *v*/*v*)) for **6** (0.006 M), DMSO for **7** (0.002 M) and aliquots of these solutions were then added in solutions (2.5 mg BSA/mL) of saline.

The experiment on the inhibitory effect of each compound of interest against the washed rabbit platelets (WRPs) aggregation was carried out as per the already described method [25]. The platelets were from male New Zealand white rabbits (about 20), individually housed in stainless steel cages in an air-conditioned room (19 ± 1 °C, 55 ± 5% humidity on a 12/12 h artificial light/dark cycle). Blood samples were drawn from the main ear artery. Microliters of PAF in a final concentration of about 10^–10^ M were added to the cuvette of the aggregometer, and the height of the platelet aggregation was monitored. The platelet aggregation induced by PAF, or thrombin was measured before (considered as 0% inhibition) and after the addition of various concentrations of the examined sample, creating a linear curve of percentage inhibition (ranging from 0 to 100%) versus different concentrations of the sample. From this curve, the concentration of the sample that inhibited 50% PAF-induced aggregation was calculated, and this value was defined as IC_50_ (Inhibitory concentration for 50% Inhibition). Dithiothreitol-insensitive PAF choline phosphotransferase, lyso-PAF:acetyl-CoA acetyltransferase and PAF-acetylhydrolase activity assays were performed on the homogenates of rabbit leukocytes [75] while the activity of lipoprotein-associated phospholipase A2 was assessed on plasma as previously described [76]. The experiments were performed in a model 400 vs. aggregometer of Chrono-Log (Havertown, PA, USA) coupled to a Chrono-Log recorder at 37 °C with constant stirring at 1200 rpm. The separation of platelets was performed with centrifugations in a 3L-R Heraeus Labofuge (Hvanau, Germany), a 400R Heraeus Labofuge and an RC-5B Sorvall (Newtown, CT, USA) refrigerated super speed centrifuge. Bovine serum albumin (BSA), PAF and analytical solvents for the biological assays were purchased from Sigma. Standard active thrombin was purchased from Sigma, St. Lewis, MO, USA. The measurements were performed in duplicates for each sample concentration in three different WRP preparations, in order to ensure reproducibility. All experiments were also followed by appropriate control tests of the solvent used (saline solution of BSA, DMSO aliquots in a saline solution of BSA-2.5 mg BSA/mL saline- or just in saline), in WRPs. In the aggregometer cuvette (250 to 500 μL) the overall amount of added DMSO, contained in the solution of the sample under study, is not higher than 2–3 μL. All results were expressed as mean ± standard deviation (SD). The *t*-test was employed to assess differences among the IC_50_ values of each metal complex against either the PAF- or thrombin-induced aggregation. Differences were statistically significant when the statistical *p*-value was smaller than 0.05. Data were analyzed using a statistical software package (SPSS for Windows, 16.0, 2007, SPSS Inc., Chicago, IL, USA) and Microsoft Excel 2007.

The HEK293T cell line (immortalized human embryonic kidney cells) was obtained from the Biomedical Research Foundation Academy of Athens (BRFAA), Athens, Greece and the HeLa cell line (cervical cancer cells) was obtained from the National Centre For Scientific Research Demokritos (NCSR Demokritos), Athens, Greece.

#### 3.3.2. Methods for Determining the Effect on the Activity of PAF Metabolic Enzymes

##### PAF-CPT Activity Assay

The assay was performed on the homogenate of leukocytes isolated as previously described [77]. Briefly, the reaction was carried out at 37 °C for 20 min in a final volume of 200 μL containing 100 mM Tris-HCl (pH 8.0), 15 mM dithiothreitol (DTT), 0.5 mM EDTA, 20 mM MgCl_2_, 1 mg/mL BSA, 100 μM CDP-Choline, 100 μΜ 1-O-alkyl-2-sn-acetyl-glycerol (AAG, added in the assay mixture in ethanol), and the sample (0.05 mg/mL final concentration of protein). The mixture of Tris, DTT, EDTA, MgCl_2_ and BSA was incubated at 37 °C for 5 min. Initially, the homogenate was added to the mixture. After 30 s, AAG was added and 30 s later the reaction was started by the addition of CDP-Choline. The reaction was stopped by adding 0.5 mL of cold methanol after 20 min.

##### Lyso PAF-AT Activity Assay

The assay was performed on the homogenate of leukocytes as previously described [78]. Briefly, the reaction was carried out at 37 °C for 30 min in a final volume of 200 μL containing 50 mM Tris-HCl (pH 7.4), 0.25 mg/mL BSA, 20 μM lyso PAF and 200 μΜ acetyl-CoA and the sample (0.125 mg/mL final concentration of protein). The reaction started with the addition of the homogenated sample and was stopped after 30 min by adding 0.5 mL of cold methanol.

##### Determination of Produced PAF by the Biosynthetic Enzymes (PAF-CPT and Lyso PAF-AT)

The extraction, purification, and determination of the produced in each assay PAF was carried out as previously described [79]. Briefly, PAF was extracted according to the Bligh–Dyer method and was separated by thin-layer chromatography (TLC) on Silica Gel G-coated plates with a development system consisting of chloroform:methanol:acetic:acid:water (100:57:16:8, *v*/*v*/*v*/*v*). PAF bands were scrapped off, and extracted and the amount of PAF was determined by the washed rabbit platelet aggregation assay. All assays were performed in triplicate and specific activities of PAF-CPT and lyso PAF-AT were expressed as pmol of produced PAF/min/mg of sample protein present in each assay.

##### Plasma PAF-AH (LpPLA_2_) Activity Assay

Plasma PAF-AH was determined in rabbit plasma by the trichloroacetic acid precipitation method using [3H] PAF as a substrate, as previously described [76]. Briefly, 2 μL of plasma were incubated with 4 nmol of [3H]-PAF (20 Bq per nmol) for 30 min at 37 °C in a final volume of 200 μL of 50 mM Tris/HCl buffer (pH 7.4). The reaction was terminated by the addition of BSA solution (final concentration 0.75 mg/mL) and followed by precipitation with trichloroacetic acid (TCA final concentration 9.6% *v*/*v*). The samples were placed in an ice bath for 30 min and subsequently centrifuged at 16,000× *g* for 5 min at 4 °C. The [3H]-acetate released into the aqueous phase was measured on a liquid scintillation counter. All assays were performed in triplicate and the enzyme-specific activity was expressed as pmol of degraded PAF/min/mg for PAF-AH of protein or µL of plasma for LpPLA_2_.

#### 3.3.3. Cell Viability Assay

##### MTT Assay

Pqx metal complexes were diluted in stock solutions of 10 mM in dimethyl sulfoxide (DMSO). A similar concentration of cisplatin dissolved in H_2_O was used. HEK293T and HeLa cells were cultured in Dulbecco’s modified Eagle’s medium (DMEM) with high glucose (Biosera, Nuaille, France) supplemented with 10% fetal bovine serum at 37 °C and 5% CO_2_. Cells were seeded in sterile tissue culture 96-well plates (SPL Life Science, Pochon, Republic of Korea) at a density of 3000 cells per well. After 24 h, the medium was removed and replaced with fresh media containing various concentrations (0.01 μΜ, 0.1 μΜ, 1 μΜ, 10 μΜ, 25 μΜ, 40 μΜ, 55 μΜ, 70 μΜ, 85 μΜ and 100 μΜ) of the compounds to be tested and was further incubated for 48 h at 37 °C. Dimethyl sulfoxide (DMSO) was used as a vehicle at ≤1%. To measure cytotoxicity, a culture medium containing the compounds (or DMSO) was removed, and 100 μL of 0.5 mg/mL MTT reagent (Applichem, Darmstadt, Germany) diluted in DMEM was added to the cells for 3 h at 37 °C. After 3 h incubation in identical conditions, MTT was removed and 200 μL of DMSO was added. Absorbance values of formazan were measured at 540 nm using a BioTek Synergy H1 microplate reader (Agilent, Santa Clara, CA, USA). All compounds were tested in triplicate. The results were statistically analyzed using the nonlinear regression by GraphPad PRISM (Version 9).

## 4. Conclusions

A series of first-row transition metal complexes bearing the 2-(2′-pyridyl)quinoxaline (pqx) ligand were biologically tested for the first time as potent inhibitors of the PAF- and thrombin-induced aggregation in washed rabbit platelets. The antiplatelet effects of the lead compounds **6** and **4** were significantly higher than those of the pqx ligand precursor and the other metal-based analogs tested. Both complexes showed moderate cytotoxicity effects in vitro, in HEK293T (human embryonic kidney cells) and HeLa cells (cervical cancer cells). In addition, the Cu-pqx (**3**) analog was the most active against the HeLa cell line, and at the same time proved to be a potent inhibitor of the PAF-induced aggregation. Consequently, the Fe-pqx (**6**) and Zn-pqx (**4**) complexes could be potentially evaluated as candidates for new antitumor agents.

The results reported herein, not only highlight the role of first-row transition metals from Cr to Zn that have been shown to be essential to life, but further reveal the importance of metal-based *inflammatory mediators* for the development of new agents with dual anti-PAF and anticancer activities. This research has emphasized the anti-PAF activities of a series of metal-based inhibitors towards the PAF-induced aggregation in WRPRs. To support our findings, the antithrombotic potency of these complexes was studied by thrombin which is a well-established thrombotic mediator and platelet agonist. The positive results, which clearly and directly show the effect of the studied compounds on the inhibition of the biological action of PAF on platelet aggregation, combined with the effect of the studied compounds on the metabolism of PAF, encourage the continuation of these studies. Further experiments on hPRPs may provide important information since the relevant conditions for the latter are closer to the in vivo ones. Accordingly, theoretical docking calculations could help us to examine if these inhibitors fit within the binding site of PAFR. This would enable our efforts to design possible antiplatelet drugs in the future.

Finally, we must point out that our research group is among the first to implicate PAF with cancer and highlight the possible role of PAF inhibitors [23] which triggered other researchers to demonstrate that the co-administration of PAF inhibitors improves the pharmacological action of anticancer drugs [64]. Recently, the biochemical mechanism by which anticancer drugs, in addition to their anticancer effect, simultaneously cause PAF production has also been reported [66]. Therefore, the co-administration with anticancer drugs and PAF inhibitors, or even better, the development of anticancer drugs with dual pharmacological action (anticancer and anti-PAF), as researched by our group, opens new avenues in the fight against cancer.

## Figures and Tables

**Table 1:** Inhibition of PAF- and thrombin-induced aggregation by complexes **1**–**7**, ligand and metal precursors towards WRPs and Thrombin.

Precursors and Metal Complexes	IC_50_ (μM) towards PAF in WRPs	IC_50_ (μM) towards Thrombin in WRPs
pqx	32 ± 15	no inhibition
*Metal precursors*		
CrCl_3_ × 6H_2_O	537 ± 59	611 ± 67
CoCl_2_ × 6H_2_O	no inhibition ^1^	no inhibition
CuCl_2_ × 2H_2_O	22 ± 3	3.23 ± 0.39 **
ZnCl_2_	30 ± 3	41.4 ± 3.7
MnCl_2_ × 4H_2_O	302 ± 30	466 ± 47
FeCl_2_ × 4H_2_O	261 ± 37	13.80 ± 1.93
NiCl_2_ × 6H_2_O	30 ± 2.7	4.90 ± 0.44
*Complexes*		
[Cr(pqx)Cl_2_(DMSO)_2_]Cl (**1**)	4.5 ± 0.9 **	54.6 ± 10.4
[Co(pqx)Cl(DMSO)]Cl (**2**)	4.1 ± 0.6 **	8.9 ± 1.3
[Cu(pqx)Cl_2_(DMSO)] (**3**)	10.6 ± 1.4	3.1 ± 0.4 **
[Zn(pqx)Cl_2_] (**4**)	3.3 ± 0.3 ***	3.5 ± 0.3 **
[Mn(pqx)Cl(DMSO)]Cl (**5**)	39 ± 6	14.0 ± 2.1
[Fe(pqx)Cl(DMSO)_3_]Cl(**6**)	1.79 ± 0.12 ***	0.46 ± 0.03 ***
[Ni(pqx)Cl(DMSO)]Cl (**7**)	6.83 ± 0.42	5.60 ± 0.34

^1^ Data obtained from ref. [60]. The symbols ** and *** stand for statistically significant differences with *p* < 0.05 and *p* < 0.005, respectively, when these metal complexes are compared with all the other ones.

**Table 2:** IC_50_ values of the pqx containing complexes **1**–**7** against the HEK293T and Hela cell lines. Cisplatin is used as a control. Values are the mean ± SD.

Compounds	HEK293T	HeLa
pqx	97.33 ± 10.46	62.46 ± 5.43
[Cr(pqx)Cl_2_(DMSO)_2_]Cl (**1**)	99.05 ± 6.16	82.84 ± 3.12
[Co(pqx)Cl(DMSO)]Cl (**2**)	67.03 ± 12.58	89.12 ± 7.77
[Cu(pqx)Cl_2_(DMSO)] (**3**)	2.10 ± 0.52	27.20 ± 1.23
[Zn(pqx)Cl_2_] (**4**)	94.92 ± 23.16	77.84 ± 6.51
[Mn(pqx)Cl(DMSO)]Cl (**5**)	28.83 ± 6.16	99.96 ± 13.18
[Fe(pqx)Cl(DMSO)_3_]Cl (**6**)	65.81 ± 13.17	70.90 ± 2.50
[Ni(pqx)Cl(DMSO)]Cl (**7**)	25.53 ± 8.61	82.34 ± 9.49
cisplatin	4.44 ± 1.29	12.77 ± 1.48

**Table 3:** Crystallographic data and structure refinement for complex (**6**).

	6
Empirical formula	C_15_H_15_N_3_OSCl_2_Cu
Molecular weight	419.80
Crystal color	yellow plate
Crystal size (mm^3^)	0.2 × 0.18 × 0.04
Temperature (K)	123.15
Crystal system	monoclinic
Space group	*P*2_1_
Unit cell dimensions	
*α* (Å)	7.6765(12)
*β* (Å)	14.246(2)
*γ* (Å)	7.6828(11)
*α* (°)	90
*β* (°)	101.405(6)
*γ* (°)	90
*V* (Å^3^)	823.6(2)
*Z*	2
*ρ*_alc_ (g cm^−3^)	1.693
*μ* (mm^−1^)	1.783
F(000)	426.0
Absorption correction	multi-scan
2*Θ* range (°)	5.41 to 58.492°
*hkl* range	−10, 10/−19, 18/−10, 10
Total data	14,946
Data/restraints/parameters	4423/205/212
refinement method	Full matrix on *F*^2^
*R*_1_, *wR*_2_	0.0431, 0.0863
*R*_1_, *wR*_2_ (all data)	0.0677, 0.0982
Min./max. density (e Å^−3^)	0.80/−1.40
Goodness-of-fit	1.078
Flack parameter	0.04(2)

## Data Availability

The data presented in this study are available on request from the corresponding author.

## Supplementary Materials

The following supporting information can be downloaded at: https://www.mdpi.com/article/10.3390/molecules28196899/s1, Figures S1–S7: FT-IR spectra of complexes **1**–**7**; Table S1: Bond lengths (Å) with estimated standard deviations for **3**; Table S2: Bond angles (°) with estimated standard deviations for **3**; Figure S8: Hydrogen bonding interactions in **3**; Figure S9: UV-Vis spectrum of **7** in DMSO; Figure S10: FT-IR spectra of **1** and **5** in DMSO; Figures S11–S18: ESI-HRMS spectra of **pqx** and complexes **1**–**7** in DMSO/methanol. Entry 21: General experimental for ESI-HRMS.

## Sample Availability

Samples of the compounds are not available from the authors.
