# Supplementary material for: First-Row Transition Metal Complexes Incorporating the 2-(2′-pyridyl)quinoxaline Ligand (pqx), as Potent Inflammatory Mediators: Cytotoxic Properties and Biological Activities against the Platelet-Activating Factor (PAF) and Thrombin
Source: Molecules. 2023 Oct 1;28(19):6899. doi: 10.3390/molecules28196899 (PMC10574351; doi:10.3390/molecules28196899)
Supplement: Supplementary file 1 [file molecules-28-06899-s001.zip › molecules-2609904-supplementary.pdf]

Supplementary material

For

First-row Transition Metal Complexes Incorporating the 2-(2'-pyridyl)quinoxaline Ligand (pqx), as Potent Inflammatory Mediator Inhibitors: Cytotoxic Properties and Biological Activities against the Platelet-Activating Factor (PAF) and Thrombin.

Antigoni Margariti [1], Vassiliki D. Papaconstantinou [2], George M. Stamatakis[2], Constantinos A. Demopoulos[2], Christina Machalia[2], Evangelia Emmanouilidou[2], Gregor Schnakenburg[3], Maria-Christina Nika[4], Nikolaos S. Thomaidis[4] and Athanassios I. Philippopoulos[1,*]

[1] Laboratory of Inorganic Chemistry, Department of Chemistry, National and Kapodistrian University of Athens, Panepistimiopolis Zografou 15771, Athens, antmargariti@gmail.com (A.M.)

[2] Laboratory of Biochemistry, Department of Chemistry, National and Kapodistrian University of Athens, Panepistimiopolis Zografou 15771; papakonstantinou.v@gmail.com (V.D.P.), stamatakisgeo@gmail.com (G.M.S.), demopoulos@chem.uoa.gr (C.A.D.), cmachali@chem.uoa.gr (C.M.), eemman@chem.uoa.gr (E.E.)

[3] Institut für Anorganische Chemie, Rheinische Friedrich-Wilhelms-Universität Bonn, Gerhard-Domagk-Straße 1, D-53121 Bonn, Germany; gschnake@uni-bonn.de (G.S.)

[4] Laboratory of Analytical Chemistry, Department of Chemistry, National and Kapodistrian University of Athens, Panepistimiopolis Zografou 15771, Athens, nikamar@chem.uoa.gr (M.N.), ntho@chem.uoa.gr (N.T.)

* Corresponding author: Prof. A. I. Philippopoulos. Tel.: +30-210-7274697; e-mail: atphilip@chem.uoa.gr

Table of Contents

Entry

1)	**Figure S1**	FT-IR (in KBr) spectrum of **1**
2)	**Figure S2**	FT-IR (ATR mode) spectrum of **2**
3)	**Figure S3**	FT-IR (in KBr) spectrum of **3**
4)	**Figure S4**	FT-IR (ATR mode) spectrum of **4**
5)	**Figure S5**	FT-IR (ATR mode) spectrum of **5**
6)	**Figure S6**	FT-IR (ATR mode) spectrum of **6**
7)	**Figure S7**	FT-IR (ATR mode) spectrum of **7**
8)	**Table S1**	Bond lengths (Å) with estimated standard deviations for **3**
9)	**Table S2**	Bond angles (°) with estimated standard deviations for **3**
10)	**Figure S8**	Hydrogen bonding interactions in complex **3**
11)	**Figure S9**	UV-Vis spectrum of **7** in DMSO
12)	**Figure S10**	FT-IR spectra of **1** and **5** in DMSO
13)	**Figure S11**	ESI-HRMS spectra of **pqx** in DMSO/methanol
14)	**Figure S12**	ESI-HRMS spectra of **1** in DMSO/methanol
15)	**Figure S13**	ESI-HRMS spectra of **2** in DMSO/methanol
16)	**Figure S14**	ESI-HRMS spectra of **3** in DMSO/methanol
17)	**Figure S15**	ESI-HRMS spectra of **4** in DMSO/methanol
18)	**Figure S16**	ESI-HRMS spectra of 5 in DMSO/methanol
19)	**Figure S17**	ESI-HRMS spectra of **6** in DMSO/methanol
20)	**Figure S18**	ESI-HRMS spectra of **7** in DMSO/methanol
21)	**General experimental for ESI-HRMS**	

1) **Fig. S1**. FTIR spectra of Cr-pqx (**1**) in KBr

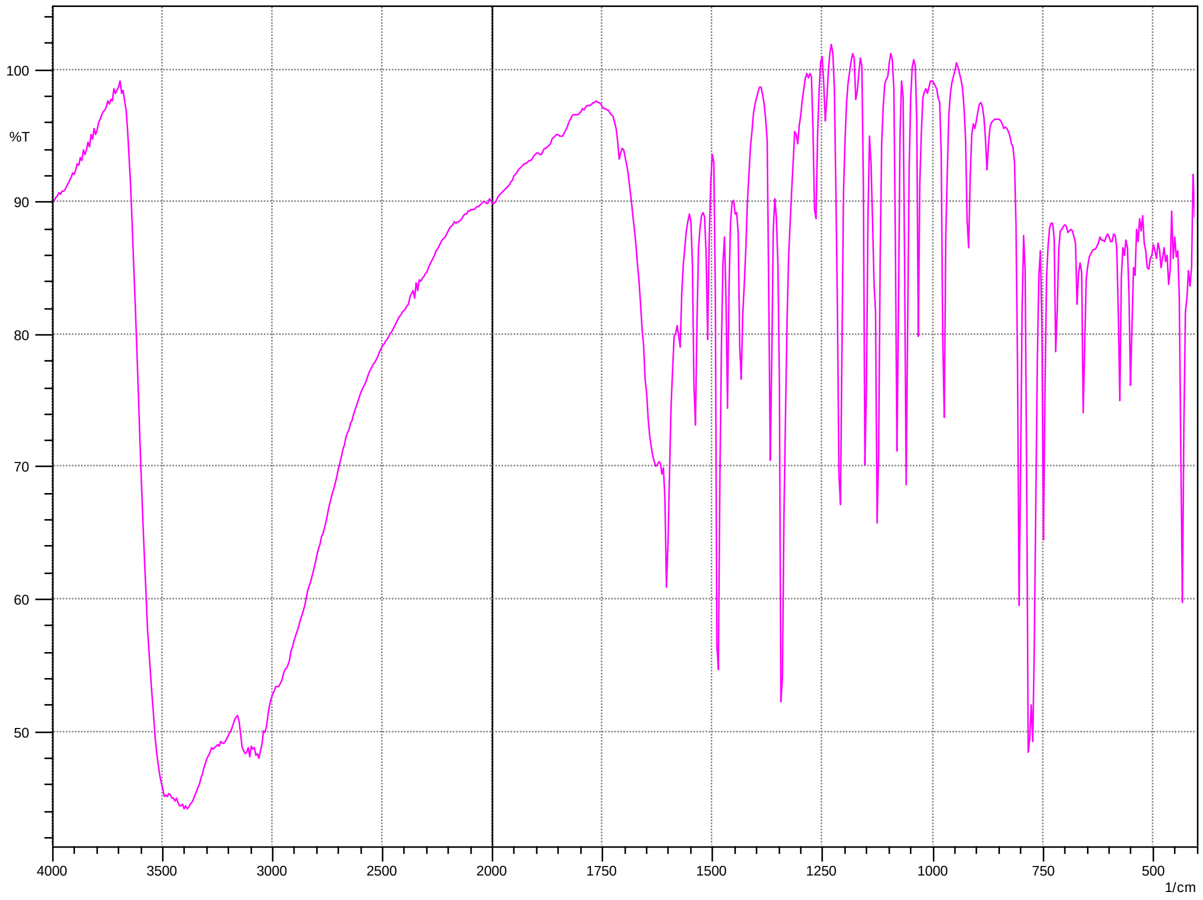

2) **Fig. S2.** ATR-FTIR spectra of Co-pqx (**2**)

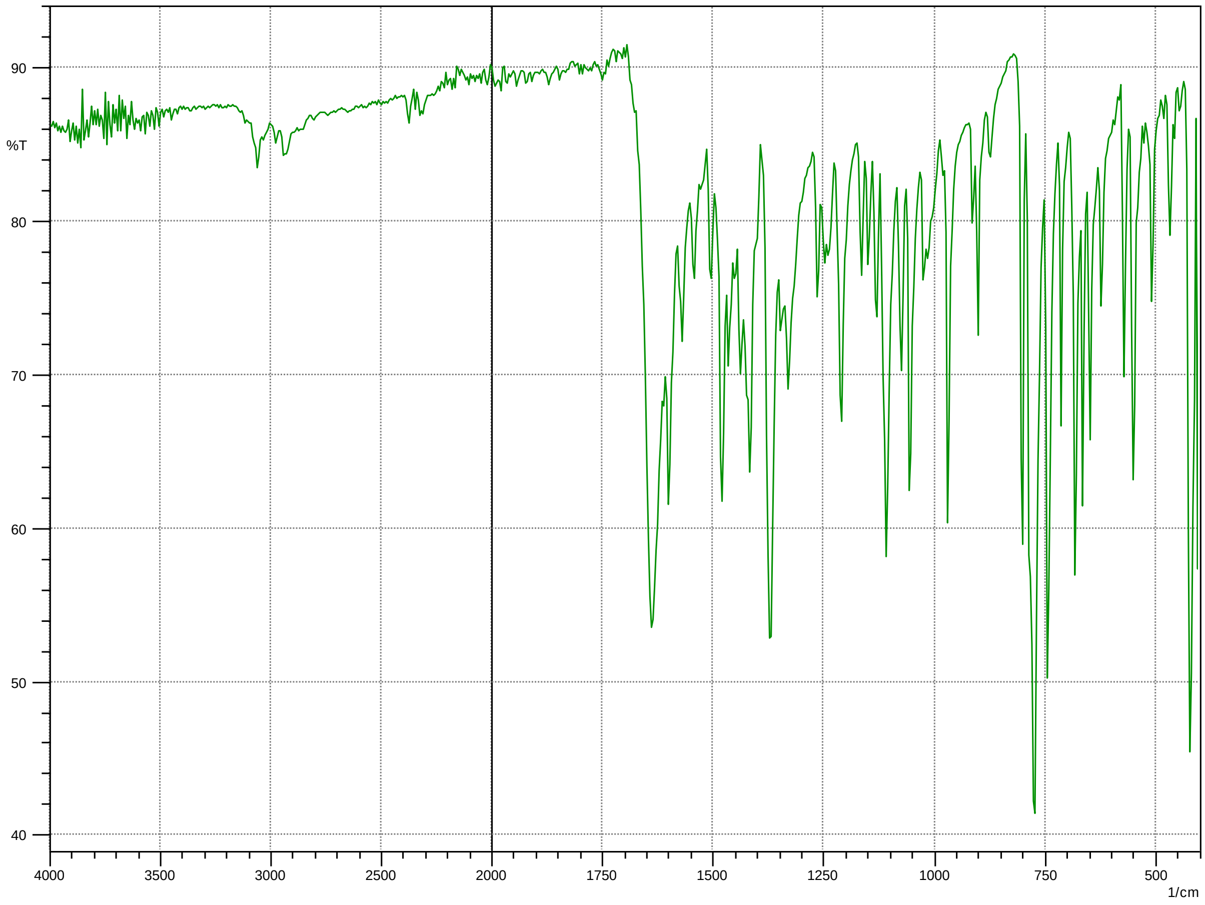

3) **Fig. S3** FTIR spectra of $CuLCl_2$(DMSO) (**3**) in KBr

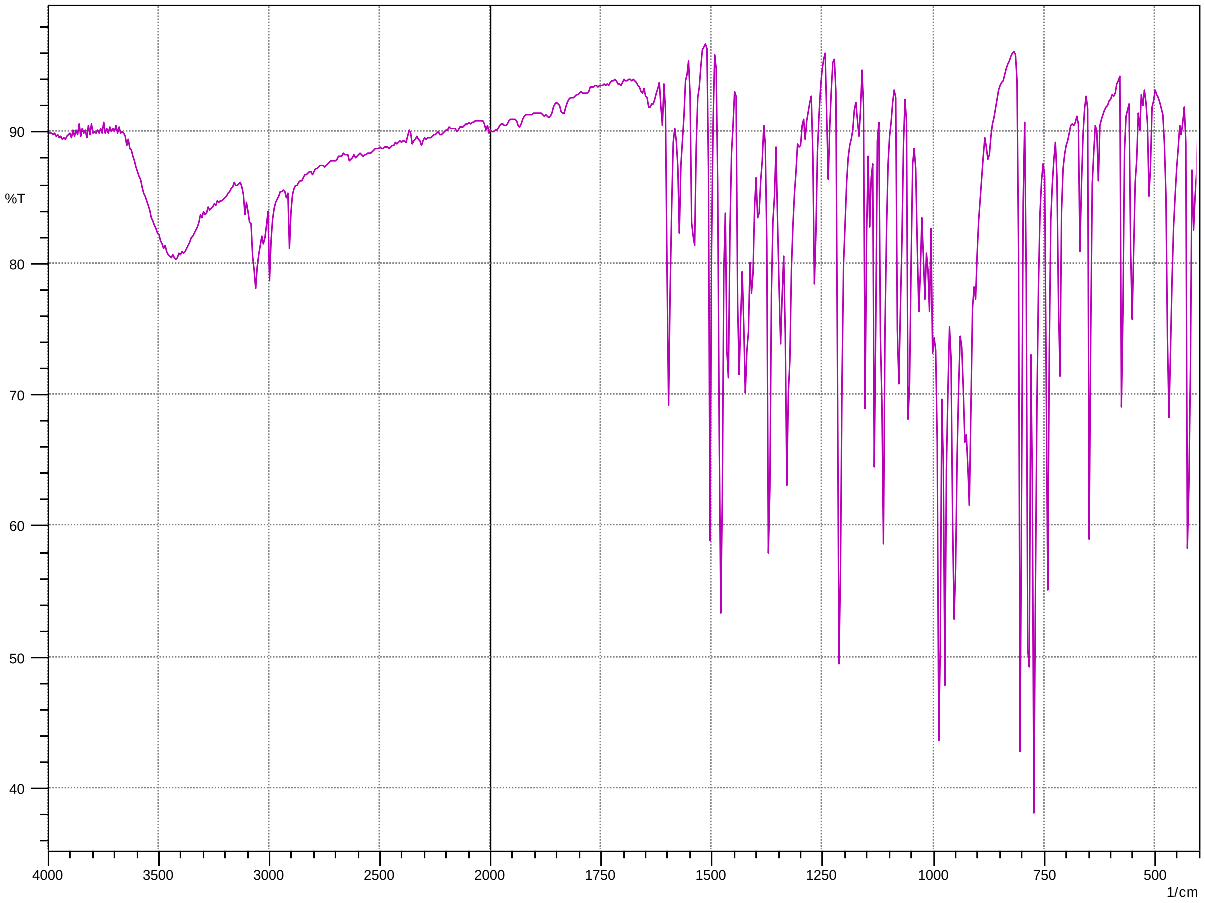

4) **Fig. S4** ATR-FTIR spectra of Zn-pqx (**4**)

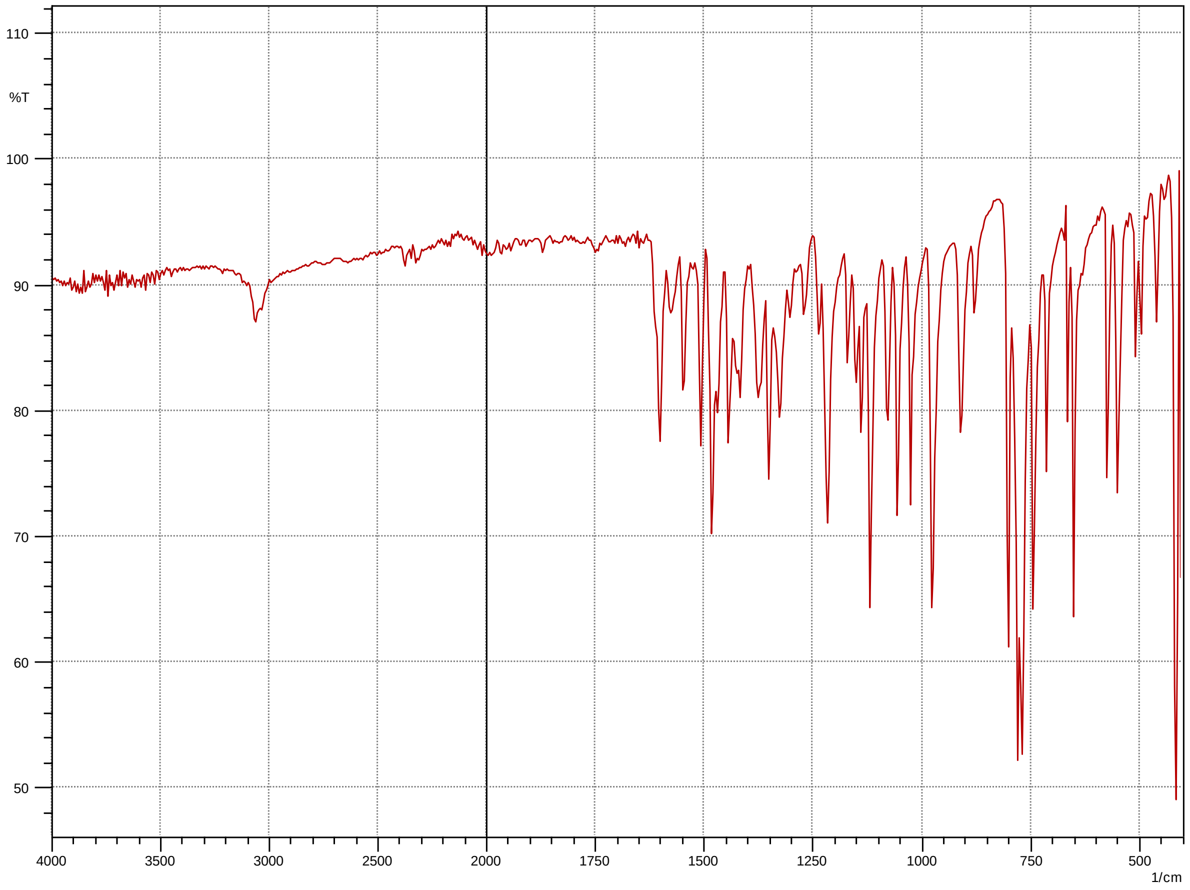

5) **Fig. S5** ATR-FTIR spectra of Mn-pqx (**5**)

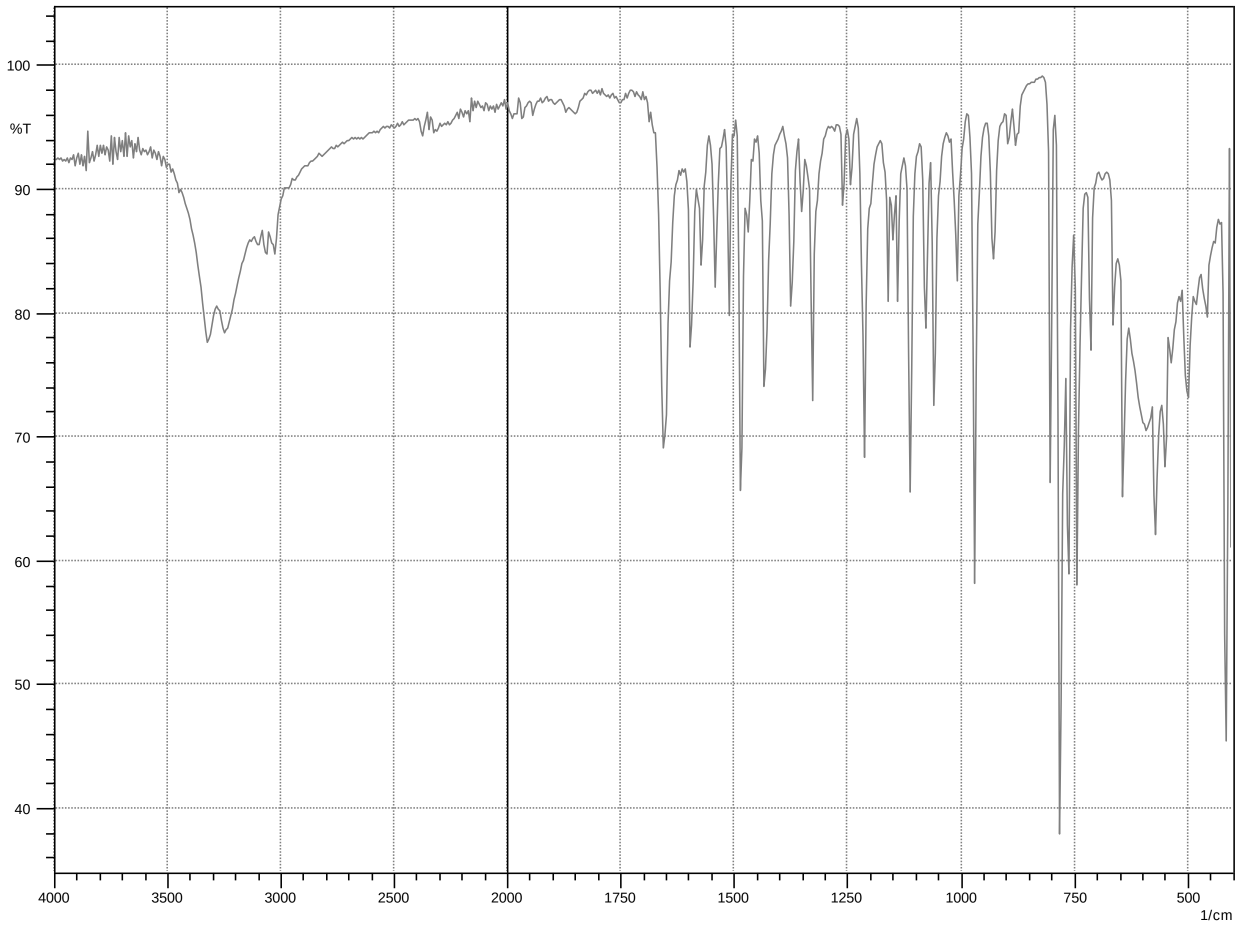

6) **Fig. S6** ATR-FTIR spectra of Fe-pqx (**6**)

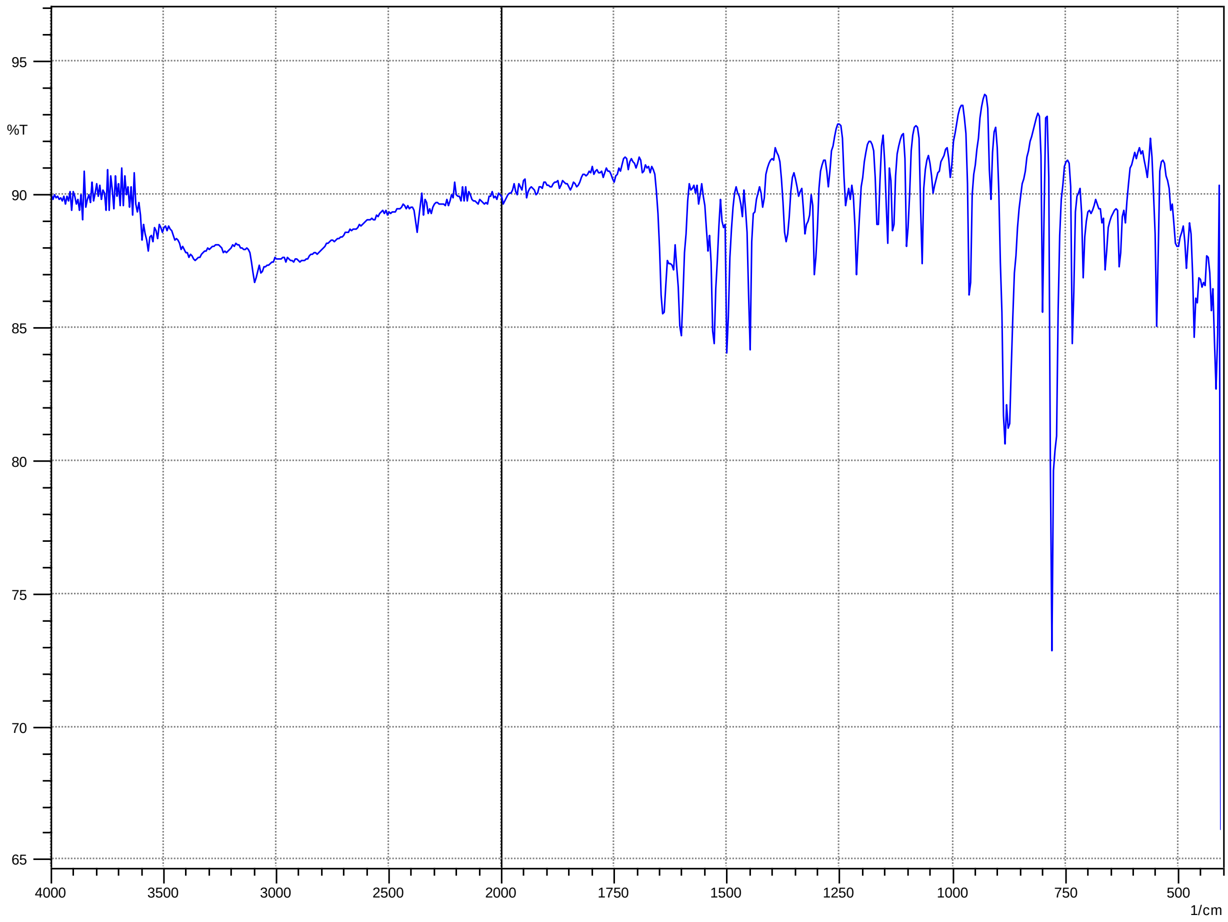

7) **Fig. S7** ATR-FTIR spectra of Ni-pqx (**7**)

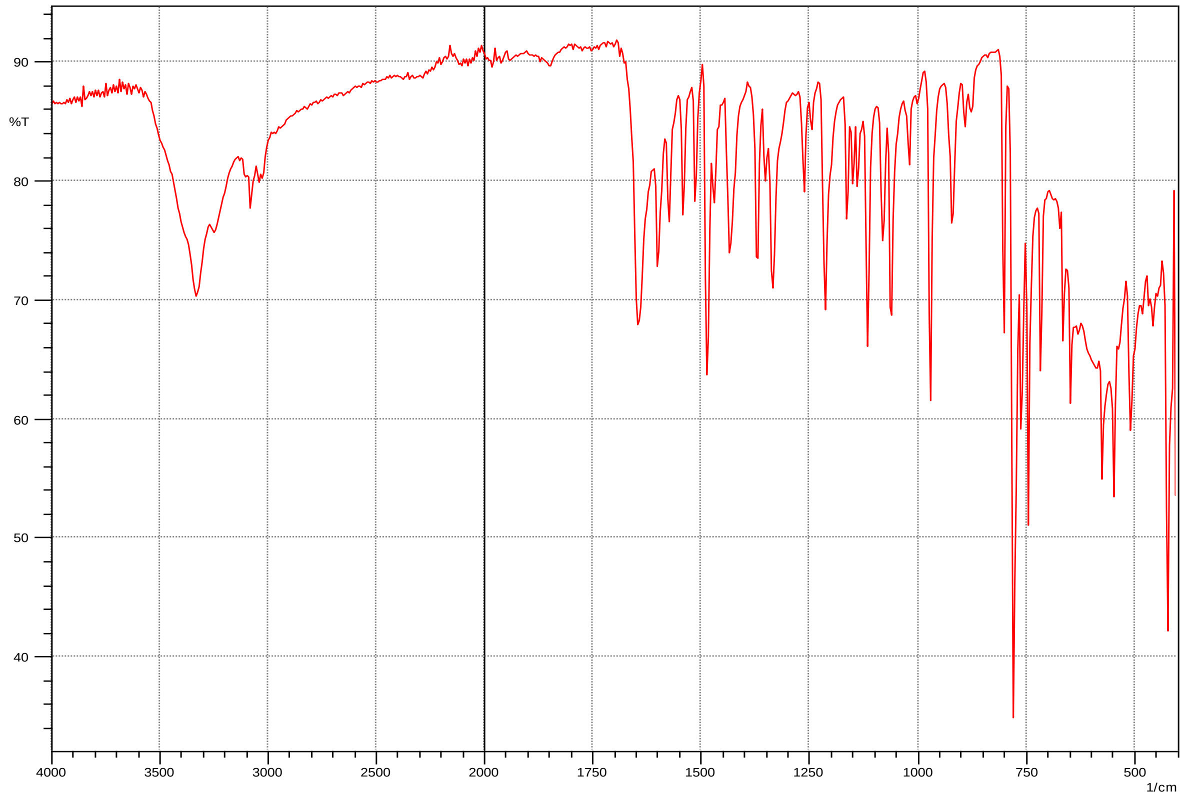

8) **Table S1**. Bond lengths (Å) with estimated standard deviations for **3**

Atom	Atom	Length/ Å	Atom	Atom	Length/Å
Cu	Cl1	2.3240(15)	N3	C9	1.333(7)
Cu	Cl2	2.3267(16)	C1	C2	1.436(8)
Cu	O	1.970(4)	C1	C5	1.460(8)
Cu	N1	2.020(5)	C3	C4	1.415(8)
Cu	N3	2.128(5)	C3	C10	1.417(8)
S	O	1.527(4)	C4	C13	1.399(9)
S	C14	1.784(6)	C5	C6	1.384(8)
S	C15	1.780(7)	C6	C7	1.382(9)
N1	C1	1.328(8)	C7	C8	1.392(8)
N1	C4	1.374(8)	C8	C9	1.375(8)
N2	C2	1.303(8)	C10	C11	1.358(9)
N2	C3	1.357(8)	C11	C12	1.405(8)
N3	C5	1.361(7)	C12	C13	1.366(9)

9) Table S2. Bond angles (°) with estimated standard deviations for **3**

Bond angles			(°)	Bond angles			(°)
N1	Cu	N3	78.88(19)	N1	C4	C3	119.0(5)
N3	Cu	Cl1	109.70(14)	N1	C4	C13	120.6(5)
N3	Cu	Cl2	115.37(13)	C13	C4	C3	120.4(5)
O	S	C14	103.9(3)	N3	C5	C1	114.2(5)
O	S	C15	105.4(3)	N3	C5	C6	121.2(5)
C15	S	C14	98.5(3)	C6	C5	C1	124.6(5)
S	O	Cu	121.5(2)	C7	C6	C5	119.9(6)
C1	N1	Cu	116.1(4)	C6	C7	C8	118.3(6)
C1	N1	C4	119.1(5)	C9	C8	C7	119.2(6)
C4	N1	Cu	124.8(4)	N3	C9	C8	122.8(6)
C2	N2	C3	117.4(5)	C11	C10	C3	119.8(6)
C5	N3	Cu	113.0(4)	C10	C11	C12	120.6(6)
C9	N3	Cu	128.2(4)	C13	C12	C11	121.3(6)
C9	N3	C5	118.7(5)	C12	C13	C4	119.0(6)

10) Figure S8. Hydrogen bonding interactions in complex **3**

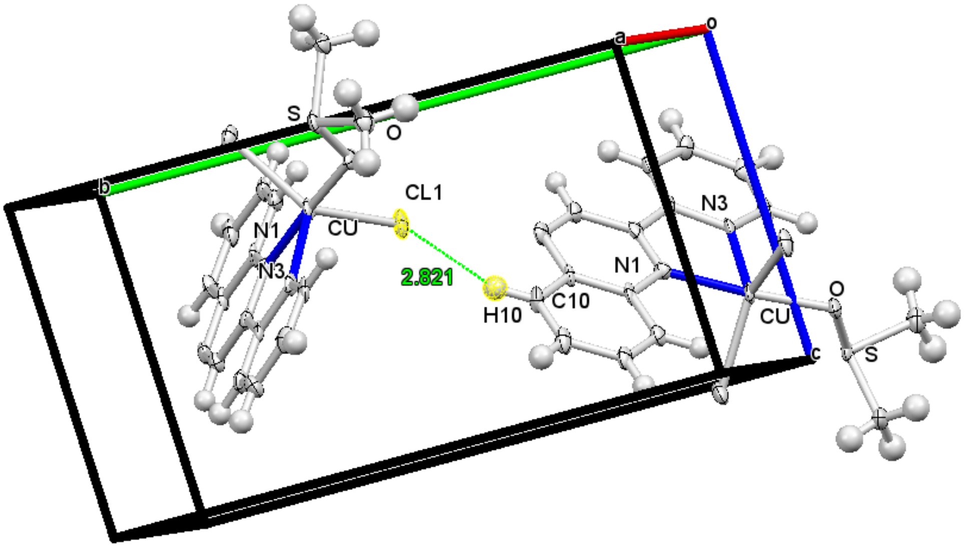

11) Figure S9. UV-Vis of complex 7

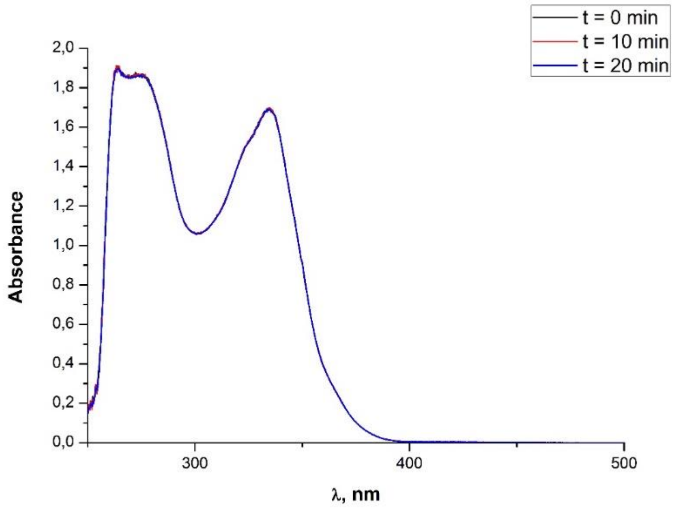

12) Figure S10. FT-IR spectra of **1** and **5** in DMSO

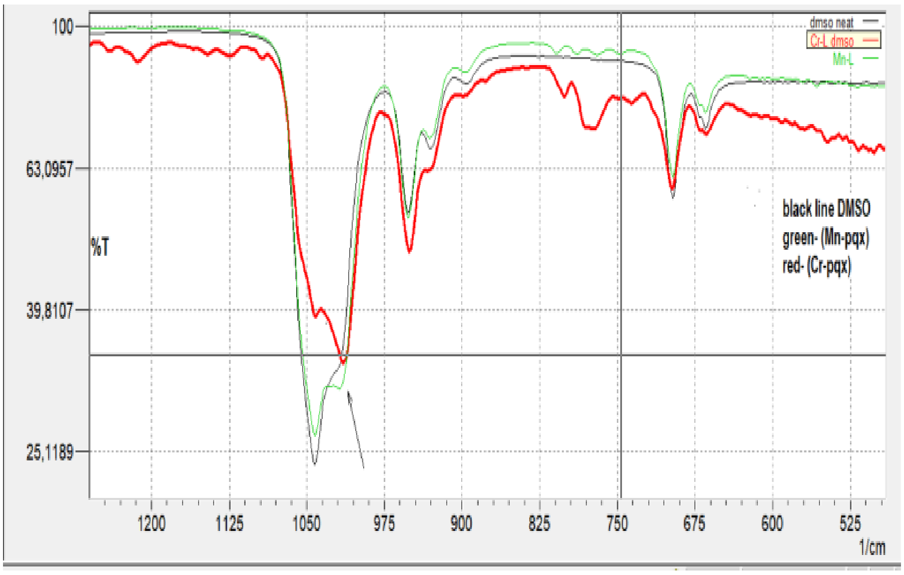

13) Figure S11. ESI-HRMS spectra of **pqx** in DMSO/methanol

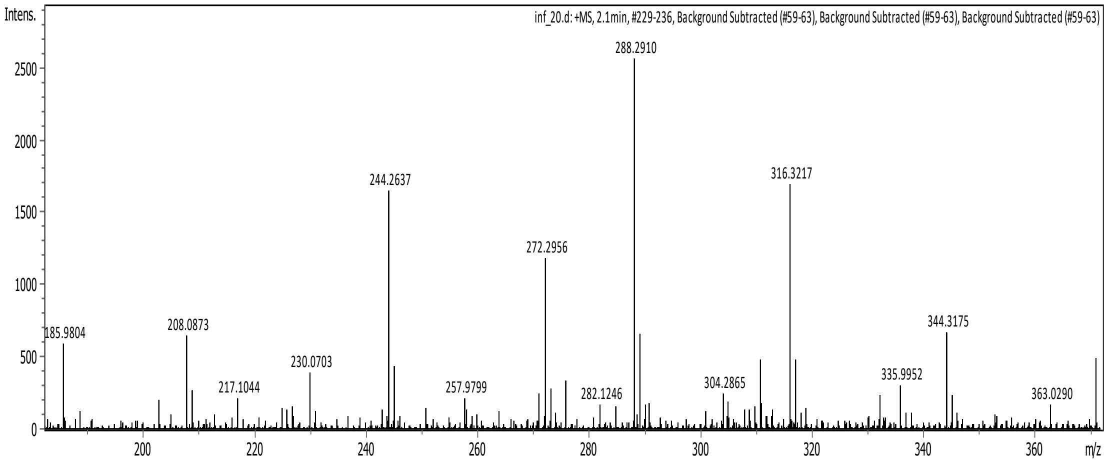

14) Figure S12. ESI-HRMS spectra of **1** in DMSO/methanol

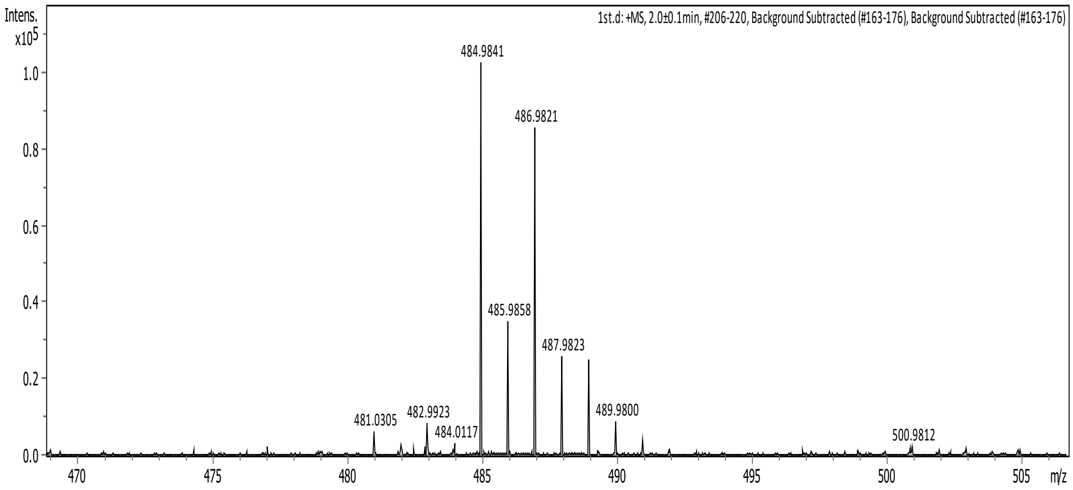

15) Figure S13. ESI-HRMS spectra of **2** in DMSO/methanol

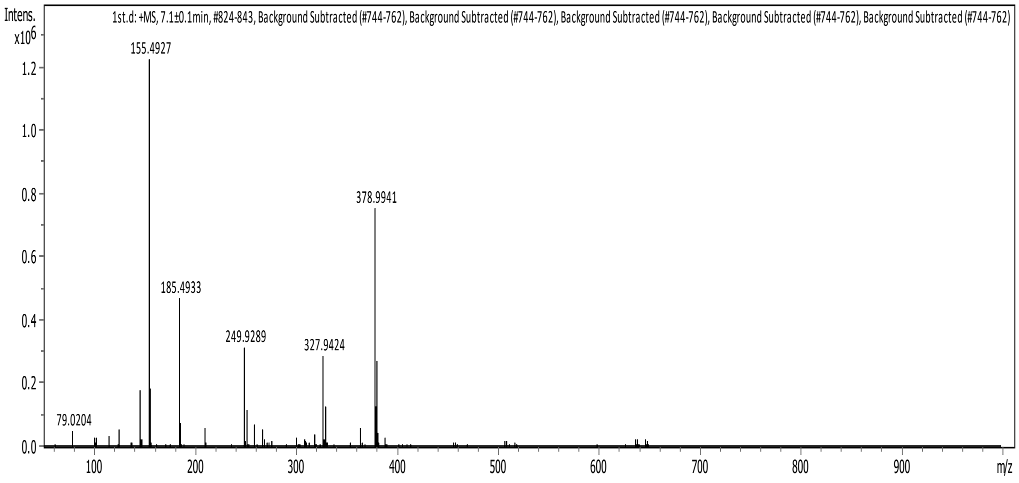

16) Figure S14. ESI-HRMS spectra of **3** in DMSO/methanol

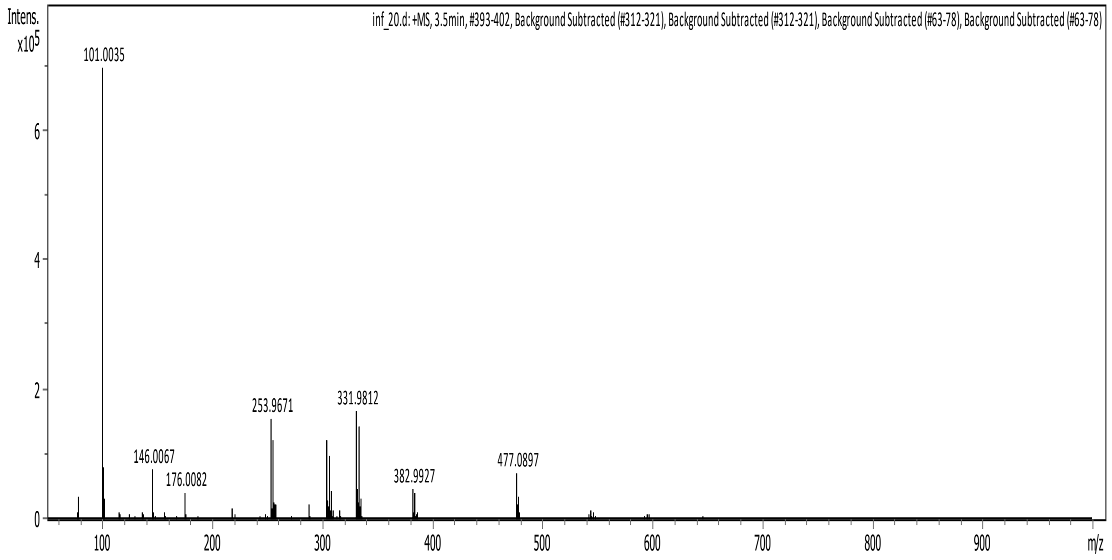

17) Figure S15. ESI-HRMS spectra of **4** in DMSO/methanol

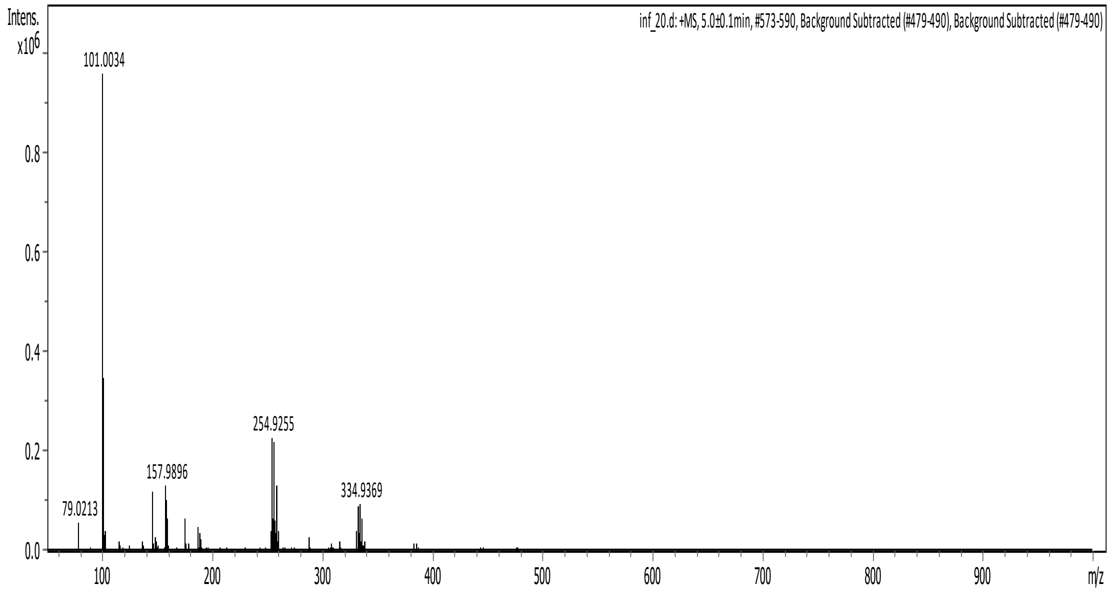

18) Figure S16. ESI-HRMS spectra of **5** in DMSO/methanol

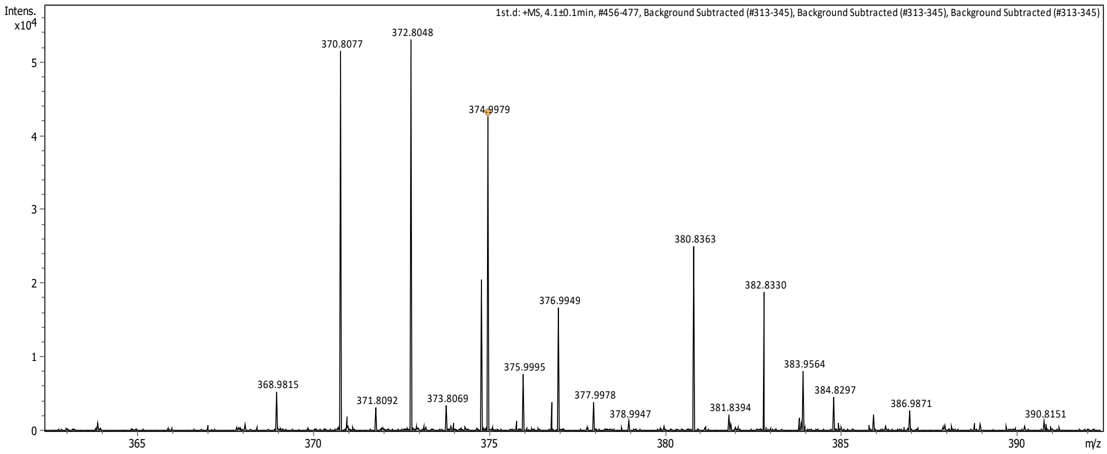

19) Figure S17. ESI-HRMS spectra of **6** in DMSO/methanol

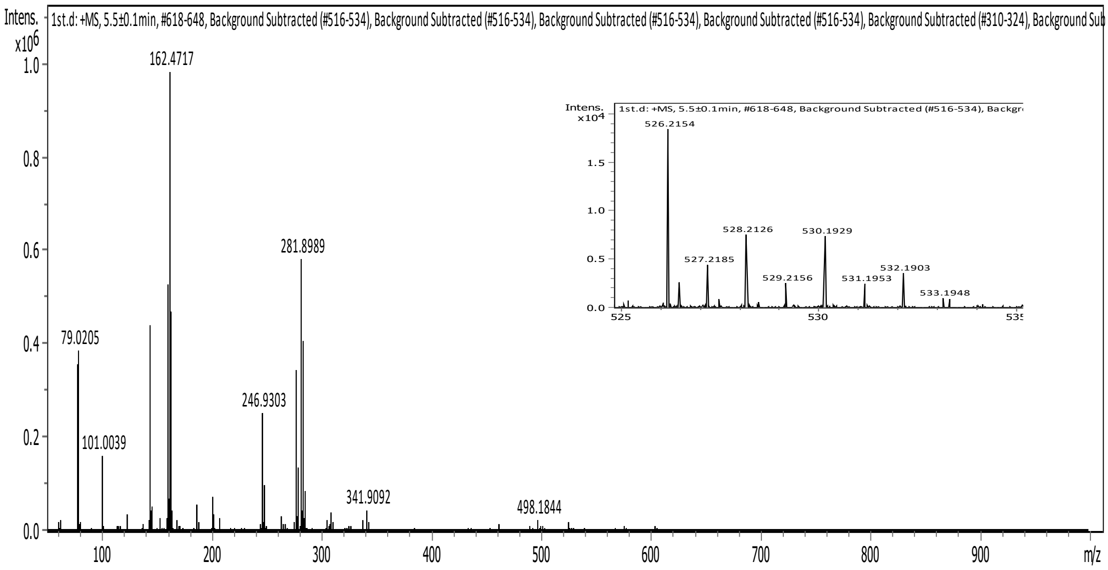

20) Figure S18. ESI-HRMS spectra of **7** in DMSO/methanol

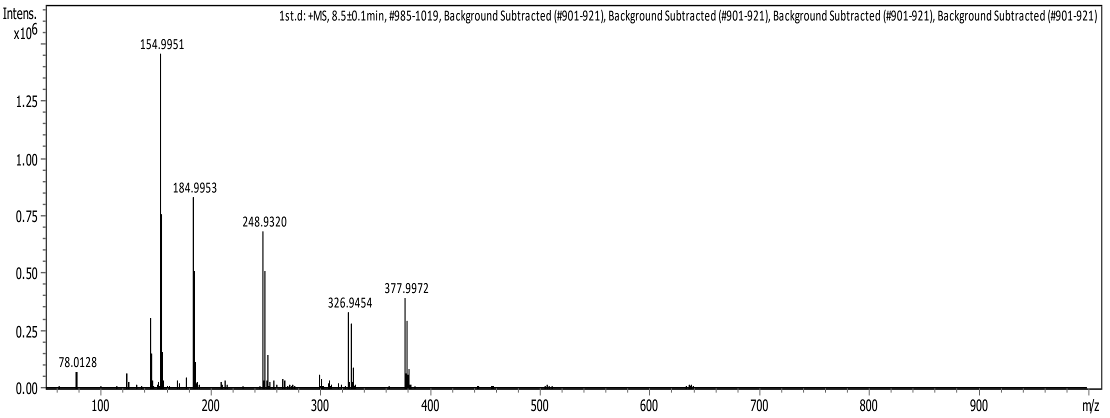

21) General experimental part for ESI-HRMS

The QToF system was equipped with an electrospray ionization interface (ESI), operating in positive ionization mode, with the following operation parameters: capillary voltage 2500 V; end plate offset −500 V; nebulizer pressure 1.0 bar; drying gas 4.0 L min^{-1}; and gas temperature 180 °C. Full scan acquisition mode with a scan rate at 1Hz was employed, recording spectra over the m/z range 50-1000. A regular system maintenance protocol was followed to assure the good operation of the HRMS system, before analysis. After cleaning the source, transfer tube and cone, the sensitivity, mass accuracy and resolution were monitored and compared to installation values. Moreover, a QToF external calibration was performed using a sodium formate solution (10 mM), producing Na $(NaCOOH)_{1-14}$ clusters over the analysis m/z range. A blank solution (mixture of Methanol/DMSO, 50/50 v/v) was also infused for subtracting any background noise from the acquired spectra. The identification relied on the mass accuracy of the pseudomolecule ion and the distinctive isotopic pattern of the formed complexes, whereas further investigation of the high-resolution mass spectrometric spectra provided insights into the mechanisms that take place under dilution with DMSO.
